# Enantioselective Flow Synthesis of Rolipram Enabled by a Telescoped Asymmetric Conjugate Addition–Oxidative Aldehyde Esterification Sequence Using *in Situ*-Generated Persulfuric Acid as Oxidant

**DOI:** 10.1021/acs.orglett.1c04300

**Published:** 2022-01-20

**Authors:** Bence S. Nagy, Patricia Llanes, Miquel A. Pericas, C. Oliver Kappe, Sándor B. Ötvös

**Affiliations:** † Institute of Chemistry, University of Graz, NAWI Graz, Heinrichstrasse 28, A-8010 Graz, Austria; ‡ Institute of Chemical Research of Catalonia (ICIQ), The Barcelona Institute of Science and Technology (BIST), Av. Països Catalans 16, E-43007 Tarragona, Spain; § Departament de Química Inorgànica i Orgànica, Universitat de Barcelona (UB), E-08028 Barcelona, Spain; ∥ Center for Continuous Flow Synthesis and Processing (CC FLOW), Research Center Pharmaceutical Engineering GmbH (RCPE), Inffeldgasse 13, A-8010 Graz, Austria

## Abstract

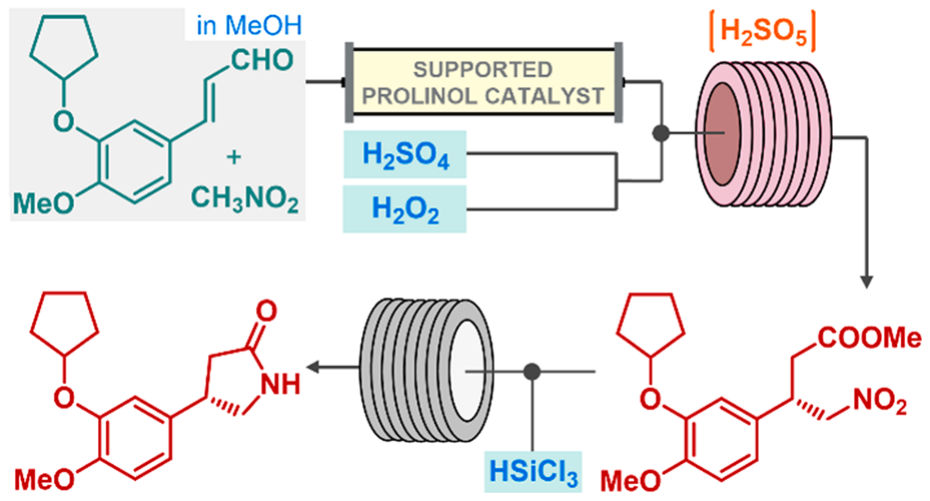

A novel approach is reported for the enantioselective flow synthesis of rolipram comprising a telescoped asymmetric conjugate addition–oxidative aldehyde esterification sequence followed by trichlorosilane-mediated nitro group reduction and concomitant lactamization. The telescoped process takes advantage of a polystyrene-supported chiral organocatalyst along with *in situ*-generated persulfuric acid as a robust and scalable oxidant for direct aldehyde esterification. This approach demonstrates significantly improved productivity compared with earlier methodologies while ensuring environmentally benign metal-free conditions.

Due to their varied biological activities as well as structural diversity, chirally branched pyrrolidones are of outstanding importance among pharmaceutically relevant heterocycles.^1^ Of these compounds, rolipram exhibits an unusually wide range of pharmaceutical effects which attracts significant attention of research.^2^ The best characterized biological activity of rolipram is the selective inhibition of the cyclic adenosine monophosphate (cAMP)-specific phosphodiesterase family known as Type IV (PDE4).^3^ It has therefore primarily been employed as an anti-inflammatory as well as antidepressant agent in numerous clinical trials^4^ but has also been reported to bear antipsychotic, antitumor, and immunosuppressive effects,^5^ and has shown potential as a treatment for multiple sclerosis.^6^ Most recently, among other PDE4 inhibitors, rolipram has actively been investigated in the treatment of COVID-19^7^-induced severe pneumonia and associated cytokine storms.^8^ Although it has often been employed as a racemate in biological studies, the pharmaceutical activity of rolipram enantiomers was found to be divergent in many cases,^3,6^ thereby necessitating enantioselective synthesis routes.

Numerous synthetic strategies have been reported that deliver single enantiomers of rolipram. Earlier methodologies utilized homochiral building blocks from the chiral pool or employ various resolution techniques, such as chiral chromatography or enzymatic resolution.^9^ Recently, enantioselective synthetic approaches have been suggested to facilitate a more direct and atom economic access to the enantiopure substance. These typically harness chiral-coordination-complex-catalyzed asymmetric hydrogenations or conjugate additions to introduce asymmetry,^10^ but metal-free organocatalysis has also proven useful in the enantioselective synthesis of rolipram.^11^ Despite the fact that enantioselective strategies exhibit remarkable benefits over classical synthetic processes, high costs of chiral catalysts, limited productivity and scalability along with the need for multiple rounds of work-up and purification as well as the difficult handling of certain chiral intermediates still significantly hamper their practical usefulness.

Despite the well-established advances of continuous flow chemistry in the multistep syntheses of active pharmaceutical ingredients (APIs),^12^ there is only one example reported for the asymmetric flow synthesis of rolipram,^13^ in which a polymer-supported chiral calcium catalyst was exploited in a nitrostyrene–malonate conjugate addition as a key step to establish asymmetry.^14^ This pioneering process afforded approximately 1 g of enantio-enriched rolipram in 1 day over four telescoped steps.

As a continuation of our ongoing interest in flow synthesis of chiral APIs and their advanced intermediates,^15^ we sought for a novel approach for the enantioselective flow synthesis of rolipram demonstrating improved productivity and scalability while ensuring environmentally reliable metal-free conditions and generating low amounts of waste. In view of the fact that only very limited asymmetric strategies are available for the direct activation of α,β-unsaturated ester substrates,^16^ the synthetic route to reach our goal relied on the enantioselective Michael-type addition of nitromethane to an appropriately substituted cinnamaldehyde derivative (**1**) in the presence of a diphenylprolinol-type organocatalyst,^17^ followed by oxidative esterification, nitro reduction, and lactamization (Scheme 1). Considering that the chiral γ-nitroaldehyde product of the Michael-type addition is labile and may decompose during workup or purification, we envisioned that a telescoped enantioselective flow synthesis of the corresponding γ-nitroester as a chiral key intermediate could be accomplished by merging the asymmetric conjugate addition with a suitable esterification protocol. Based on a step-economy-driven process-design, we were aiming for a direct oxidative approach for ester formation, thereby eliminating the need for the generation of the free carboxylic acid intermediate.^18^

**Scheme 1:**
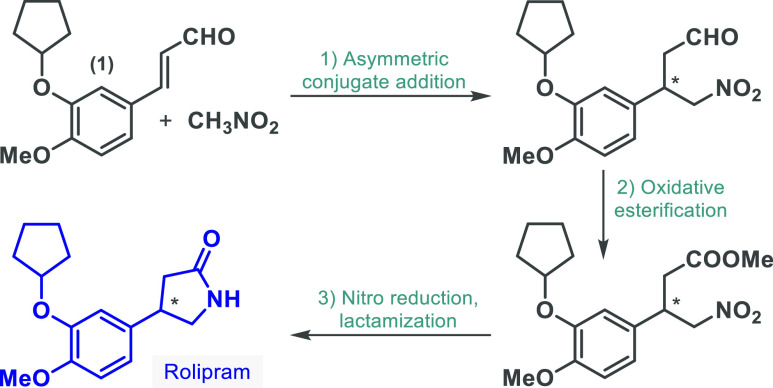
Enantioselective Synthetic Strategy

Taking into account practical and environmental aspects,^19^ we selected a cross-linked polystyrene-supported *cis*-4-hydroxydiphenylprolinol *tert*-butyldimethylsilyl (TBS) ether as a chiral organocatalyst (**2**) for the enantioselective conjugate addition.^20^ In our earlier studies,^15^**2** was proven as a robust, reusable, and leaching-free catalyst that would possibly enable the telescoping of the asymmetric key step. A simple flow setup was therefore established by using a heated Omnifit glass column (10 mm ID, adjustable height) filled with 1.0 g of the immobilized catalyst (*f* = 0.445 mmol g^–1^). The conjugate addition was performed by pumping a neat mixture of aldehyde **1** and 5 equivalents (eq) of nitromethane at 65 °C. Gratifyingly, at 75 μL min^–1^ flow rate (corresponding to 15 min residence time) and in the presence of 0.6 eq of acetic acid as additive, 95% of **1** was chemoselectively converted to furnish the corresponding γ-nitroaldehyde (**3**) with an excellent ee of 94% (Scheme 2). Importantly, conversion was found to decrease notably at higher flow rates or by performing the reaction at room temperature. Furthermore, a reduction of the nitromethane or the acetic acid amount exhibited a similar negative effect on the reaction outcome.

**Scheme 2:**
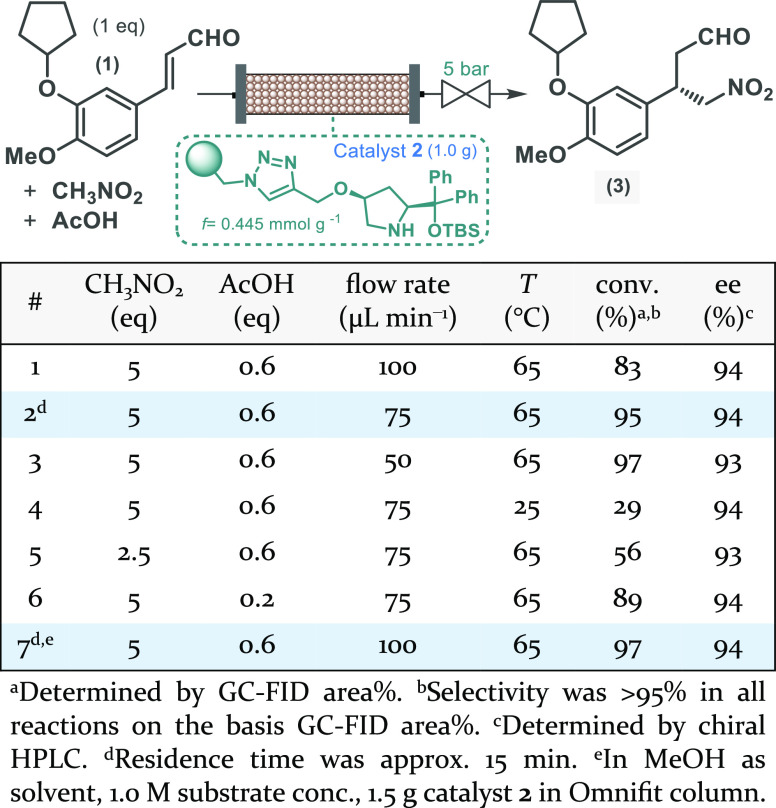
Effect of Reaction Conditions on the Organocatalytic Flow Synthesis of γ-Nitroaldehyde **3**

With a reliable flow process for the enantioselective synthesis of chiral γ-nitroaldehyde **3** in hand, the next step was to establish a suitable method for the subsequent ester formation. For this, we sought for a simple and easily scalable protocol that is, most importantly, compatible with the organocatalytic conjugate addition in a telescoped process. The direct oxidative esterification of aldehydes is widely achieved in the presence of various homogeneous or heterogeneous transition metal catalysts.^21^ However, considering that such methodologies typically involve costly ligands, insoluble catalysts, as well as long reaction times, and in the case of heterogeneous catalytic sources uncontrollable leaching issues may also arise, we did not consider transition-metal-catalyzed approaches. Although metal-free strategies utilizing *N*-heterocyclic carbene-based catalysts have also been reported for oxidative ester formations,^22^ these were not considered either due to the relatively high costs and the accompanying incompatibility issues with larger scale operations.

Inspired by our earlier results on continuous flow aldehyde to carboxylic acid oxidations,^15a,23^ we initially attempted the oxidative esterification by using *in situ*-generated performic acid as the oxidant in the presence of MeOH as the alcohol component. Unfortunately, the selectivity toward the desired ester was very low, and the reaction furnished the corresponding carboxylic acid as the major product along with numerous further side products (see the Supporting Information for details).

Peroxymonosulfuric acid (or persulfuric acid) is a powerful oxidizing agent with well-established applications in industrial scale wastewater treatment, such as purification of cyanide containing effluents of gold processing plants.^24^ Given the fact that, during handling and storage, its tendency toward explosive decomposition entails a considerable safety risk,^25^ persulfuric acid is typically manufactured on site using concentrated (cc) H_2_SO_4_ as a stable precursor in the presence of H_2_O_2_.^26^ As a consequence of its difficult handling and hazardous nature, the synthetic usefulness of persulfuric acid in organic chemistry remained largely underexplored.^27^ Considering that continuous flow reactors are well-suited for the safe generation of highly reactive reagents,^28^ we anticipated that, by means of *in situ* formation and concomitant consumption within a closed continuous flow environment, safety hazards could be minimized and persulfuric acid could be exploited as a cost-efficient and scalable oxidant for the direct oxidative ester formation.^29^ In order to test our hypothesis, hydrocinnamaldehyde as a simple model substrate together with H_2_SO_4_ and 35 wt % aq H_2_O_2_, both in MeOH as solvent, were pumped as separate feeds, and the combined mixture was directed through a heated reaction coil where simultaneous persulfuric acid generation and oxidative esterification took place (Scheme 3A). Since sulfonic peracids were shown to have potential for the selective oxidation of various substances,^30^*p*-toluenesulfonic peracid generated following the same strategy was also investigated as an oxidizing agent in the direct ester formation (Scheme 3A). To our delight, at 100 °C and 30 min residence time, both *in situ*-generated reagents ensured high selectivity toward oxidative esterification with only small amounts of carboxylic acid **5** as the only side product. The corresponding dimethyl acetal (**6**), which is known as an intermediate during oxidative ester formation from aldehydes,^18^ was detected only in the case of lower reaction temperature or shorter residence time. Importantly, in the case of the persulfuric acid-mediated process, sufficiently pure ester could be achieved by simple extractive workup of the quenched reaction mixture, whereas, for the quantitative removal of residual *p*-toluenesulfonic acid, chromatographic purification was required. Therefore, oxidative esterification of chiral γ-nitroaldehyde **3** was next attempted in the presence of *in situ*-generated persulfuric acid, and following fine-tuning of the most important reaction conditions, the corresponding nitroester (**7**) was quantitatively and selectively accessed (Scheme 3B; see also Scheme S9 and Figure S2 in the Supporting Information).

**Scheme 3:**
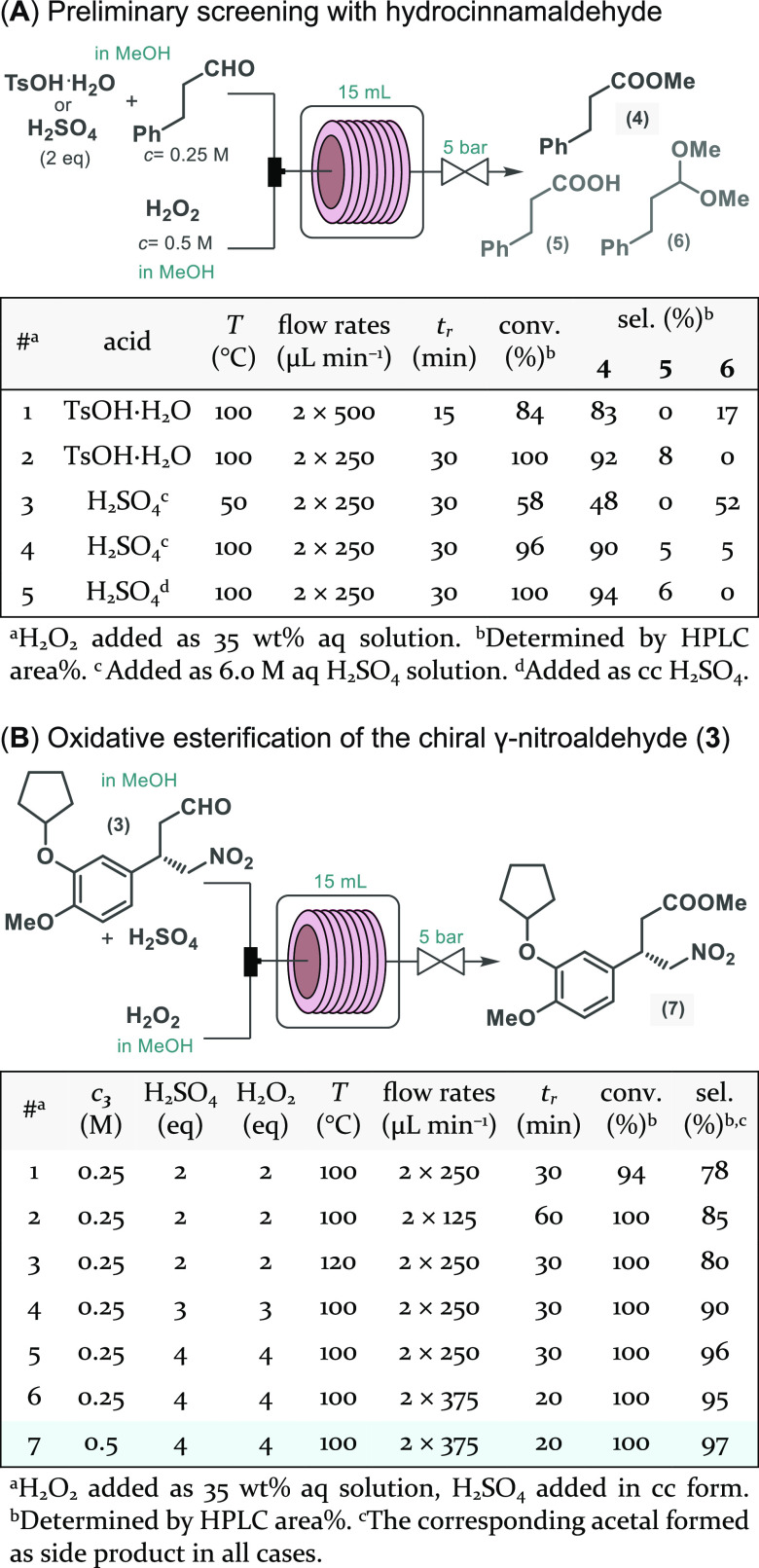
Continuous Flow Oxidative Esterifications Using *in Situ*-Generated Peracids

In order to achieve the chiral γ-nitroester (**7**) without isolation of the nitroaldehyde intermediate (**3**), we next wanted to combine the organocatalytic enantioselective conjugate addition with the subsequent oxidative esterification into an uninterrupted flow sequence. To ensure the compatibility of the reaction steps prior to telescoping, the conjugate addition was repeated in a MeOH solution containing 1.0 M aldehyde **1** together with 5 eq of CH_3_NO_2_ and 0.6 eq of acetic acid (Scheme 2), and the oxidative esterification was repeated using a modified three-feed setup to separately introduce MeOH solutions of all three components involved (Scheme S10 in the Supporting Information). Besides these modifications, all other reaction parameters were set to the previously optimized values, and the individual reaction segments were simply merged using two Y-mixers and a three-port valve. The substrate stream exiting the organocatalyst column was first combined with a 4.0 M H_2_SO_4_ feed and then with a 2.0 M H_2_O_2_ feed, both at flow rates that corresponded to 4 eq with respect to the aldehyde stream. The resulting mixture was finally directed through a heated coil where simultaneous persulfuric acid generation and oxidative aldehyde esterification took place (Scheme 4). The telescoped system was run for 3 h under steady state conditions ensuring 5.23 g (86% yield, 1.74 g h^–1^ productivity) of the key nitroester intermediate in a sufficiently pure form after extractive workup. Importantly, the ee of the product was 94%, and the process generated only a small amount of waste, as demonstrated by an *E*-factor of 9.3.

**Scheme 4:**
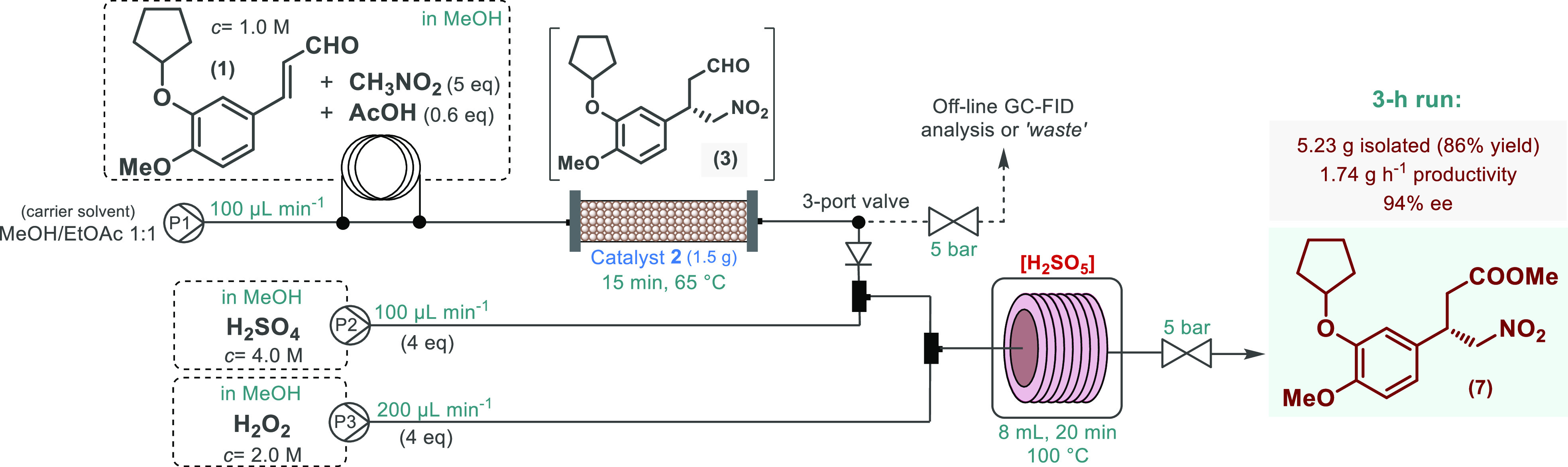
Continuous Flow Enantioselective Synthesis of the Chiral Key Intermediate of Rolipram via a Telescoped Asymmetric Conjugate Addition–Oxidative Esterification Sequence

To complete the flow synthesis of rolipram, a suitable method for nitro reduction and concomitant lactamization was needed. Due to the incompatibility of the telescoped conjugate addition–oxidative esterification sequence with the nitro reduction, an interrupted process was targeted. In most known protocols for rolipram synthesis, similar reductions are achieved by means of heterogeneous catalytic hydrogenations.^10,11,13^ Nevertheless, we instead attempted a trichlorosilane-mediated approach in order to ensure metal-free conditions and to eliminate gas handling.^31^ For this purpose, γ-nitroester **7** and trichlorosilane, both dissolved in dry CH_2_Cl_2_, were mixed in a Y-piece and the resultant stream was passed through a residence time coil at room temperature before being quenched in aq NaOH solution (Scheme 5). The substrate solution also contained an excess amount of *N*,*N*-diisopropylethylamine (DIEA) required for the generation of the actual dichlorosilylene reducing species.^32^ Although, the reaction performed well in CH_2_Cl_2_, a solvent switch was attempted to environmentally more acceptable CH_3_CN. For the trichlorosilane stream, pure CH_3_CN proved sufficient, but for the substrate/DIEA stream, CH_3_CN/CH_2_Cl_2_ 7:1 was used to ensure a single-phase solution. Further parameter optimization was conducted to find out that 4 eq of DIEA and 4 eq of trichlorosilane were necessary to achieve quantitative and selective nitro reduction/lactamization within 10 min residence time. Finally, a 4 h long run was performed under the previously optimized reaction conditions to attain 1.10 g (83% yield) of (*S*)-rolipram with 94% ee.

**Scheme 5:**
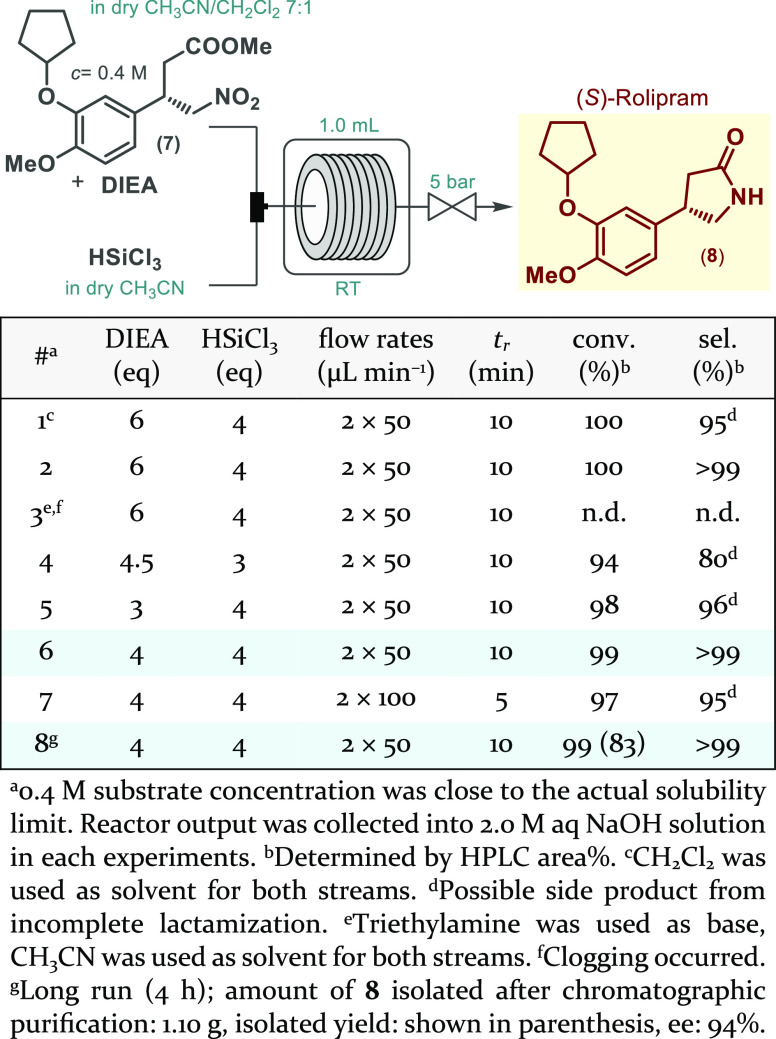
Synthesis of (*S*)-Rolipram via Continuous Flow Metal-Free Nitro Reduction/Lactamization of **7**

In summary, a three-step process has been reported for the enantioselective flow synthesis of rolipram. To access key intermediate **7** directly from the appropriate cinnamaldehyde derivative (**1**), a telescoped asymmetric conjugate addition–oxidative esterification sequence was developed. The asymmetric conjugate addition component of this reaction sequence was achieved using a resin supported *cis*-4-hydroxydiphenylprolinol organocatalyst, while the subsequent direct aldehyde esterification was accomplished by employing persulfuric acid as an effective oxidizing agent. With the purpose of minimizing safety hazards and ensuring facile scalability, persulfuric acid was generated *in situ* from H_2_SO_4_ as a stable precursor. The telescoped organocatalytic conjugate addition–oxidative esterification flow sequence yielded key nitroester intermediate **7** in a sufficiently pure form after extractive workup and ensured a productivity of 1.74 g h^–1^. Finally, rolipram was synthesized by metal-free nitro reduction/lactamization in the presence of trichlorosilane under mild conditions.

## Supporting Information Available

Synthetic procedures, additional reaction data, compound characterization data, copies of NMR spectra, and HPLC chromatograms (PDF)
