# Supplementary material for: Enantioselective Flow Synthesis of Rolipram Enabled by a Telescoped Asymmetric Conjugate Addition–Oxidative Aldehyde Esterification Sequence Using in Situ-Generated Persulfuric Acid as Oxidant
Source: Org Lett. 2022 Jan 20;24(4):1066–71. doi: 10.1021/acs.orglett.1c04300 (PMC8822492; doi:10.1021/acs.orglett.1c04300)
Supplement: Supplementary file 1 — ol1c04300_si_001.pdf [file ol1c04300_si_001.pdf]

SUPPORTING INFORMATION

for

Enantioselective Flow Synthesis of Rolipram Enabled by a Telescoped Asymmetric Conjugate Addition−Oxidative Aldehyde Esterification Sequence using *in Situ*-Generated Persulfuric Acid as Oxidant

Bence S. Nagy,[a] Patricia Llanes,[b] Miquel A. Pericas,[b,c] C. Oliver Kappe*,[a,d] and Sándor B. Ötvös*,[a,d]

[a]Institute of Chemistry, University of Graz, NAWI Graz, A-8010 Graz, Austria

[b]Institute of Chemical Research of Catalonia (ICIQ), The Barcelona Institute of Science and Technology (BIST), E-43007 Tarragona, Spain

[c]Departament de Química Inorgànica i Orgànica, Universitat de Barcelona (UB), E-08028 Barcelona, Spain

[d]Center for Continuous Flow Synthesis and Processing (CC FLOW), Research Center Pharmaceutical Engineering GmbH (RCPE), A-8010 Graz, Austria

*E-mail: oliver.kappe@uni-graz.at (COK); sandor.oetvoes@uni-graz.at (SBÖ)

Table of Contents

1. General information S2
2. Synthesis of catalyst **2** S3
3. Synthesis of compound **1** S4
4. Experimental procedure for the organocatalytic conjugate addition S5
5. Studies towards the oxidative esterification of chiral aldehyde **3** S5
  5.1. Initial attempt with *in situ*-generated performic acid S5
  5.2. Oxidative esterification with *in situ*-generated *p*-toluenesulfonic peracid and persulfuric acid S7
6. Experimental procedure for the telescoped flow synthesis of chiral ester **7** S9
7. Experimental procedure for the nitro reduction/lactamization S11
8. Preparation of racemic reference samples S11
9. Characterization data S12
10. Collection of NMR spectra and HPLC chromatograms S13
11. References S21

1. General information

All solvents and chemicals were obtained from typical commercial vendors and were used as received, without any further purification.

Column chromatographic purification was performed by using a Biotage Isolera automated flash chromatography system with cartridges packed with KP-SIL, 60 Å (32–63 μm particle size). Analytical thin-layer chromatography (TLC) was carried out using Merck silica gel 60 GF254 plates. Compounds were visualized by means of UV or by using $KMnO_4$.

^{1}H- and ^{13}C-NMR spectra were recorded on a Bruker Avance III 300 MHz instrument at room temperature, in $CDCl_3$ as solvent, at 300 MHz and 75 MHz, respectively. Chemical shifts (δ) are reported in ppm using TMS as internal standard. Coupling constants are given in Hz units.

GC-FID analysis was performed on a Shimadzu GCFID 2030 instrument equipped with a flame ionization detector, using an RTX-5MS column (30 m × 0.25 mm ID × 0.25 μm) and helium as carrier gas (40 cm s^{-1} linear velocity). The injector temperature was set to 280 °C. After 1 min at 50 °C, the temperature was increased by 25 °C min^{-1} to reach 300 °C and then kept constant at 300 °C for 3 min. The detector gases used for flame ionization were hydrogen and synthetic air (5.0 purity).

Analytical HPLC measurements were carried out on a C18 reversed-phase column (150 × 4.6 mm, particle size 5 mm) at 37 °C using mobile phases A [H_2O/CH_3CN 90:10 (v/v) + 0.1% TFA] and B (CH_3CN + 0.1% TFA) at a flow rate of 1.5 mL min^{-1}. The gradient applied was as follows: linear increase from 30% solution B to 100% B in 8 min, hold at 100% solution B for 2 min.

The ee of the compounds was determined by using a Shimadzu HPLC system (DGU-14A degasser, SCL-10A VP system controller, SPD-10 UV-VIS detector, LC-20AT pumps) and Chiralpak® IA or IB chiral columns with isocratic mixtures of hexane and *i*PrOH as eluent. Chromatographic conditions are represented in section 9.

Optical rotation was measured in $CHCl_3$ (HPLC-grade) at 25 °C against the sodium D-line (λ= 589 nm) on a Perkin Elmer Polarimeter 341 using a 10-cm pathlength cell.

High resolution mass spectra were recorded in either negative or positive mode on an Agilent 6230 TOF LC/MS (G6230B) by flow injections on an Agilent 1260 Infinity Series HPLC (HiP Degasser G4225A, Binary Pump G1312B, ALS Autosampler G1329B, TCC Column thermostat G1316A, DAD Detector G4212B).

IR spectra were recorded on a Bruker Tensor 27 / Diamond ATR FT-IR spectrometer. Elemental analysis of the immobilized organocatalyst was performed on a LECO CHNS 932 micro-analyzer.

Equipment for the continuous flow reactions was assembled using commercially available components. Liquid streams were pumped by using Syrris® Asia syringe pumps. Flow systems were pressurized by using an adjustable backpressure regulator (BPR) from Zaiput and/or by using a fixed-pressure BPR from IDEX. Fillable reactor columns were heated by using a Syrris® Asia column heater. Reaction coils were heated by means of a conventional oil bath. Reagent feeds were either streamed directly or by using injection valves and sample loops. Sample loops and reactor coils were made by using perfluoroalkoxy alkane (PFA) tubings (1/16” OD, 0.80 mm ID or 1/8” OD, 1.58 mm ID). Details of reaction setups as well as general procedures can be found in sections 4–7.

The E-factor was calculated by dividing the mass of waste generated by the mass of product formed.

CAUTION: Sulfuric acid is highly corrosive causing rapid tissue destruction and serious chemical burns. Persulfuric acid is one of the strongest oxidants known. It is unstable and potentially explosive, especially in mixtures with organic substances. Extreme care must therefore be taken when handling these substances! All equipment must be set up in a well-ventilated fume hood and personal protective equipment must be worn during experimentation. A thorough safety assessment should be made before conducting any experiments.

2. Synthesis of catalyst 2

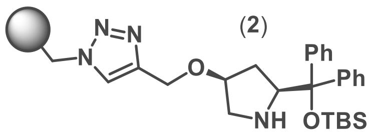

Catalyst **2** was immobilized on a cross-linked polystyrene resin (100-200 mesh) through a 1,2,3-triazole linker. The monomer synthesis and the azide–alkyne cycloaddition-based immobilization was carried out according to our recently published procedure.[S1,S2]

The level of functionalization of the polystyrene-supported catalyst *f* (mmol of monomeric catalyst / gram of resin) was calculated based on the results of nitrogen elemental analysis by the following formula:[S3]

f (mmol g^{-1}) = %N × 1000 × (number of N atoms)$^{-1}$ × M_W(N)$^{-1}$ × 100^{-1}

Elemental analysis: N 2.49, C 82.90, H 7.46

f= 0.445 mmol g^{-1} (Complete functionalization.)

The heterogeneous material was characterized by means of IR (ATR):

v= 3060, 3025, 2923, 2851, 1601, 1492, 1451, 1251, 1065, 1026, 834, 755, 695, 542 cm^{-1}

The characterization of the material matches the data reported in the literature.[S4,S5]

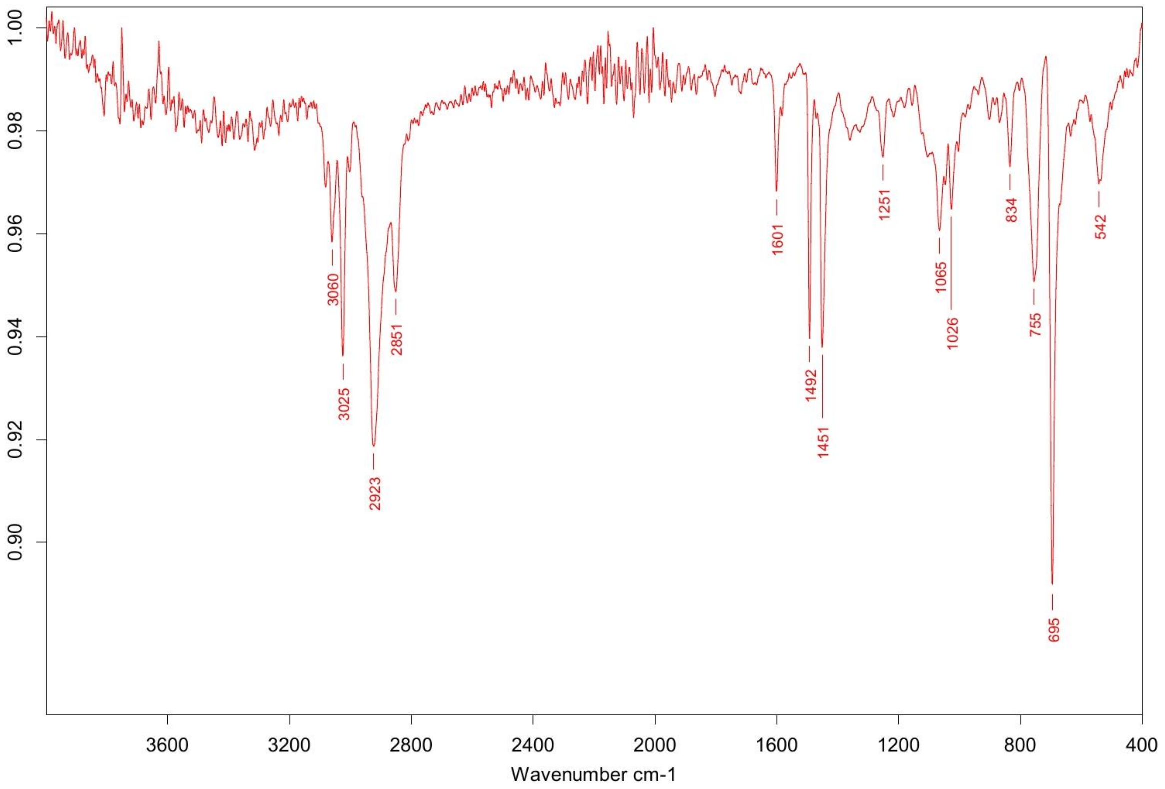

Figure S1. FT-IR (ATR) spectrum of immobilized organocatalyst **2**.

3. Synthesis of compound 1

Compound **1** was prepared according to procedures reported in the literature.[S6–S8]

To a solution of guaiacol (**1a**) (1 eq, 0.04 mol, 5.0 g) in CH_3CN (50 mL) at ambient temperature, trifluoroacetic anhydride (TFAA; 1.1 eq, 6.2 mL) was added. The mixture was stirred for 5 min, then a solution of *t*BuOK (1.0 M in *t*BuOH; 0.1 eq, 4.0 mL) was added slowly. The resultant mixture was stirred for 45 min. A solution of *N*-bromosuccinimide (NBS; 1.1 eq, 7.83 g) in CH_3CN (50 mL) was added slowly *via* an addition funnel. The orange mixture was stirred for 24 h, then the solvent was removed by evaporation and the obtained residue was suspended in CH_2Cl_2 (50 mL). A 6 N aq solution of NaOH (20 ml) was added, and the layers were separated. The aqueous layer was acidified with cc HCl until pH 2 was reached and was extracted with CH_2Cl_2. After being separated, the organic layer was washed with brine, dried over $MgSO_4$ and concentrated on a rotary evaporator to afford **1b** (7.4 g) as a reddish oil in 92% yield. NMR data of the compound matches the reported literature.[S9]

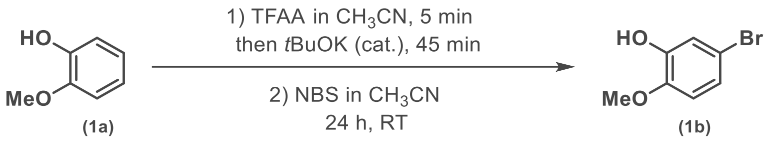

Scheme S1. Synthesis of compound **1b**.

To a mixture consisting of **1b** (1 eq, 36.1 mol, 7.3 g), K_2CO_3 (72.1 mmol, 9.95 g) and $Bu_4N^+Br^-$ (3.62 mmol, 1.17 g) in THF (110 mL) as solvent, cyclopentyl bromide (65.6 mmol, 6.64 mL) was added, and the resulting material was stirred at 75 °C for 18 h. After that, the reaction mixture was cooled to room temperature, filtered through a pad of celite and concentrated on a rotary evaporator. The crude product was diluted with EtOAc and washed with water (3 × 200 mL). The organic layer was dried over Na_2SO_4, filtered and concentrated on a rotary evaporator to give 9.55 g (98%) of **1c** as a brown oil. NMR data of the compound matches the reported literature.[S10]

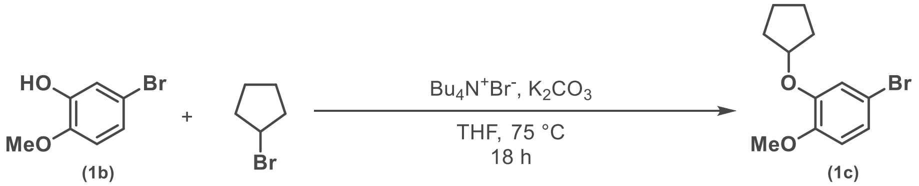

Scheme S2. Synthesis of compound **1c**.

To a stirred solution of **1c** (1 eq, 34.6 mmol, 9.35 g) in DMF (140 mL), acrolein diethyl acetal (103.9 mmol, 15.8 mL), Bu_4NOAc (≥90% purity; 69.3 mmol, 23.2 g), K_2CO_3 (51.9 mmol, 7.20 g), KCl (34.6 mmol, 2.6 g) and $Pd(OAc)_2$ (233 mg, 1.04 mmol) were added. The mixture was stirred for 18 h at 90 °C. After cooling to room temperature, 2 N HCl was added slowly, and the reaction mixture was stirred for 10 min at the same temperature. The resulting material was diluted with diethyl ether and washed with water (3 × 200 mL). The combined organic layers were washed with brine, dried over Na_2SO_4, filtered and concentrated on a rotary evaporator. The crude product was purified by means of column chromatography using a mixture of EtOAc/cyclohexane as eluent to give 7.75 g (91%) of **1** as a beige solid. The material was characterized by means of GC-MS and NMR techniques. The NMR data of the compound matches the reported literature.[S11]

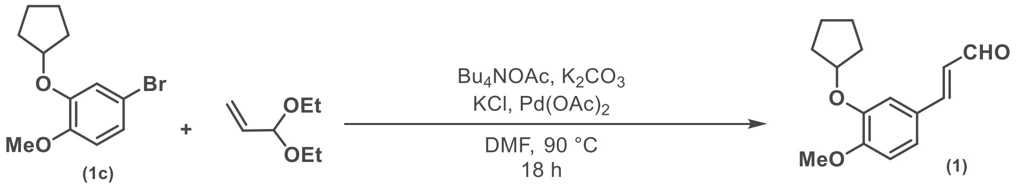

Scheme S3. Synthesis of compound **1**.

The synthesis of **1** was repeated on multiple batches.

4. Experimental procedure for the organocatalytic conjugate addition

1.0 g of catalyst **2** was loaded into an adjustable Omnifit® glass column (10 mm ID) which was heated by a Syrris® Asia column heater. Prior to the reactions, the catalyst bed was swollen by pumping EtOAc at 200 µL min^{-1} for 30 min. The system was pressurized at 5 bar by using a Zaiput BPR. With EtOAc serving as carrier solvent, the reaction mixture consisting of α,β-unsaturated aldehyde **1** (1 eq), CH_3NO_2 (2.5 or 5 eq) and AcOH (0.2 or 0.6 eq) was streamed by using a Syrris® Asia syringe pump equipped with an injection valve and a sample loop (4 mL; 1/16" OD, 0.80 mm ID). (EtOAc was chosen as pre-swelling and carrier solvent due to its environmentally-benign nature and also because it swells the catalyst carrier similarly to CH_3NO_2.) In In each run, the product stream was collected for 5 min after reaching steady state. Between each experiment, the catalyst bed was washed with EtOAc/AcOH 9:1 (0.5 mL min^{-1}, 20 min). Excess CH_3NO_2 and residual AcOH was removed by evaporation and the resulting material was analyzed by means of GC-FID and chiral HPLC. (See Scheme 2 in the manuscript for the corresponding data.)

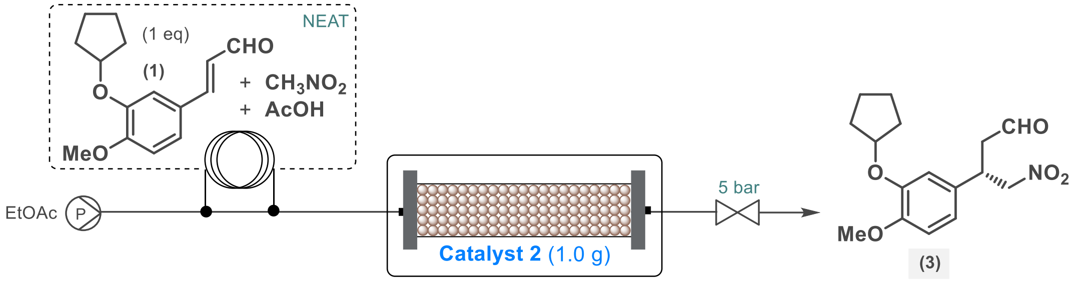

Scheme S4. Continuous flow setup for the organocatalytic conjugate addition between aldehyde **1** and CH_3NO_2.

In order to enable telescoping with the subsequent oxidative aldehyde esterification, the conditions of the organocatalytic conjugate addition were modified as follows. The reaction mixture consisting of **1** (1.0 M, 1 eq), CH_3NO_2 (5 eq) and AcOH (0.6 eq) was prepared in MeOH as solvent. By using the same setup as shown in Scheme S4, the reaction mixture was fed at a flow rate of 100 µL min^{-1} (4-mL sample loop, 1/16" OD, 0.80 mm ID). The catalyst bed (adjustable Omnifit® glass column, 10 mm ID) encompassed 1.5 g of catalyst **2** and was heated at 65 °C. Under these conditions, the residence time was determined experimentally as 15 min. The product stream was collected for 5 min after reaching steady state, then excess CH_3NO_2 and residual AcOH was removed by evaporation and the resulting material was analyzed by means of GC-FID and chiral HPLC.

5. Studies towards the oxidative esterification of chiral aldehyde 3

5.1. Initial attempt with in situ-generated performic acid

On the basis of our earlier procedure developed for sustainable aldehyde to carboxylic acid oxidations under flow conditions,[S12] oxidative esterification of a simple model substrate, hydrocinnamaldehyde was attempted by using *in situ*-formed performic acid as oxidant in the presence of MeOH as alcohol component. Performic acid was continuously generated from formic acid and hydrogen peroxide and was consumed immediately within the closed environment of a simple reactor coil.

A typical procedure for the experiment is as follows.

A 0.5 M solution of hydrocinnamaldehyde in HCOOH/MeOH 1:1 and 35 wt% aq. H_2O_2 solution were pumped as separate streams (P1 and P2; at 475 µL min^{-1} and 20.3 µL min^{-1}, respectively; corresponds to 1 eq of both reagents) by using Syrris® Asia syringe pumps equipped with two injection valves and two sample loops (SL1: 20 mL and SL2: 1.5 mL; 1/16" OD, 0.80 mm ID, each). With MeOH serving as carrier solvent, the feeds were combined in a Y-mixer. The resulting stream was directed through a 15-mL reaction coil (1/16" OD, 0.80 mm ID; 30 min residence time) which was heated at 100 °C in an oil bath. The system was pressurized at 5 bar by using a Zaiput BPR. The product stream was collected for 5 min after reaching steady state. The resulting material was analyzed by reversed-phase analytical HPLC, and also by ^{1}H NMR after evaporation. The results of the experiment are shown in Table S1.

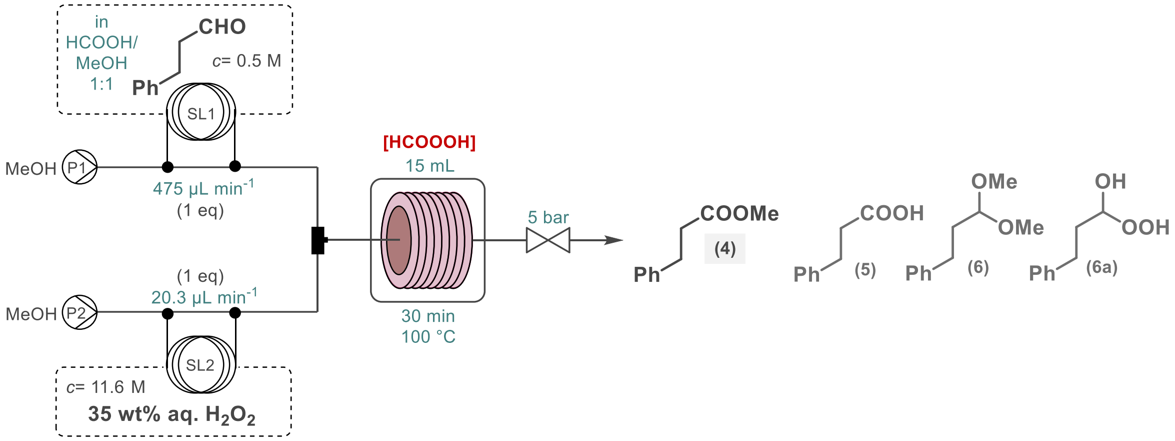

Scheme S5. Continuous flow setup for the oxidative esterification of hydrocinnamaldehyde with *in situ*-generated performic acid.

Table S1. Results of the flow reaction shown in Scheme S5.

#	Conversion (%)[a]	Chemoselectivity (%)[a]				
		**4**	**5**	**6**	**6a**	Unidentified[b]
1	95	13	37	8	10	32

[a]Determined by HPLC area%. [b]Unidentified side products. These substances may involve, for example, the corresponding hydroperoxyacetal and *gem*-dihydroperoxide.

In order to assign HPLC signals of side products **6** and **6a**, reference samples were prepared according to the following procedures. (Hydrocinnamic acid, **5** is commercially available.)

*Side product **6**:* A 0.5 M solution of hydrocinnamaldehyde in MeOH was pumped at a flow rate of 500 µL min^{-1} by using a Syrris® Asia syringe pump equipped with an injection valve and a sample loop (20 mL; 1/16" OD, 0.80 mm ID) with MeOH serving as carrier solvent. The stream was directed through a 15-mL reaction coil (1/16" OD, 0.80 mm ID; 30 min residence time) which was heated at 120 °C in an oil bath. The system was pressurized at 5 bar by using a Zaiput BPR. The product stream was collected for 10 min after reaching steady state. The resulting material was analyzed by reversed-phase analytical HPLC, and also by ^{1}H NMR after evaporation. The experiment yielded a reference sample of side product **6** in >95% purity without any further work-up or purification. NMR data of the compound matches the reported literature.[S13]

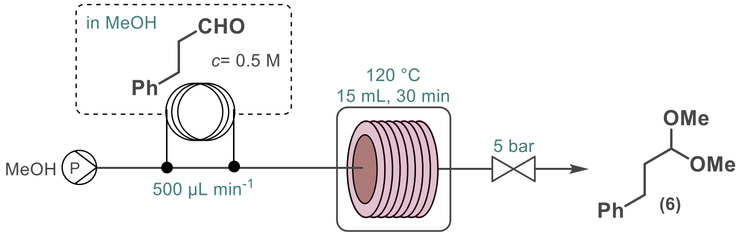

Scheme S6. Synthesis of side product **6**.

*Side product **6a**:* A 0.5 M solution of hydrocinnamaldehyde in MeOH and 0.5 M solution of H_2O_2 (prepared from 35 wt% aq. H_2O_2) were pumped as separate streams (P1 and P2; at 300 µL min^{-1} and 450 µL min^{-1}, respectively; corresponds to 1 eq of hydrocinnamaldehyde and 1.5 eq of H_2O_2) by using Syrris® Asia syringe pumps equipped with two injection valves and two sample loops (SL1: 15 mL and SL2: 20 mL; 1/16" OD, 0.80 mm ID, each). With MeOH serving as carrier solvent, the feeds were combined in a Y-mixer. The resulting stream was directed through a 15-mL reaction coil (1/16" OD, 0.80 mm ID; 20 min residence time) which was heated at 80 °C in an oil bath. The system was pressurized at 5 bar by using a Zaiput BPR. The product stream was collected for 10 min after reaching steady state. The resulting material was analyzed by reversed-phase analytical HPLC, and also by ^{1}H NMR after

evaporation. The experiment yielded a reference sample of side product **6a** in >95% purity without any further work-up or purification. NMR data of the compound matches the reported literature.[S14]

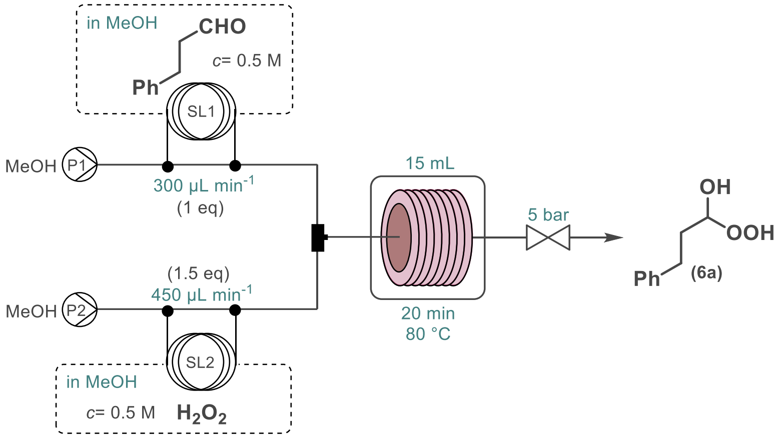

Scheme S7. Synthesis of side product **6a**.

5.2. Oxidative esterification with in situ-generated p-toluenesulfonic peracid and persulfuric acid

Initial screening was performed with hydrocinnamaldehyde as model substrate, then oxidative esterification of aldehyde **3** was attempted using the same setup. (See Scheme 3 in the manuscript for the corresponding reaction data.)

A typical procedure for the experiments is as follows.

Substrate solution containing hydrocinnamaldehyde or aldehyde **3** (1 eq, 0.25 or 0.5 M) together with TsOH·H_2O or H_2SO_4 (2, 3 or 4 eq; H_2SO_4 was used as 6.0 M aq solution or in cc form) in MeOH and a solution of H_2O_2 (0.5, 0.75 or 1.0 M; prepared from 35 wt% aq. H_2O_2) in MeOH were pumped as separate streams (P1 and P2) by using Syrris® Asia syringe pumps equipped with two injection valves and two sample loops (SL1 and SL2: 5, 10 or 15 mL; 1/16" OD, 0.80 mm ID, each). With MeOH serving as carrier solvent, the feeds were combined in a Y-mixer. The resulting stream was directed through a 15-mL reaction coil (1/16" OD, 0.80 mm ID; 15, 20, 30 or 60 min residence time) which was heated at 50, 100 or 120 °C in an oil bath. The system was pressurized at 5 bar by using a Zaiput BPR. The reactor outlet was directed into a flask containing a stirred mixture of saturated aq. $NaHCO_3$ and some MnO_2 in order to quench any excess oxidant. The product stream was collected for 5 min after reaching steady state. Then, the resulting mixture was extracted with CH_2Cl_2, washed with brine and dried over $MgSO_4$. After evaporation, the obtained material was analyzed by reversed-phase analytical HPLC. To ensure that no incidental carboxylic acid side product was lost during the quenching procedure, in some instances approx. 20 µL aliquot of the crude reactor outlet was diluted directly with 1 mL of CH_3CN/H_2O 9:1 and was analyzed by HPLC.

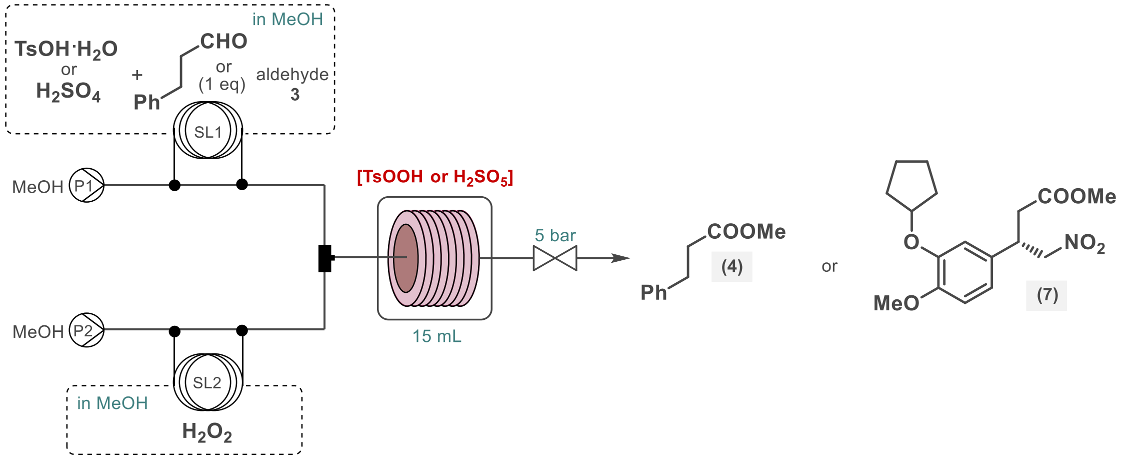

Scheme S8. Continuous flow setup for oxidative esterifications with *in situ*-generated *p*-toluenesulfonic peracid or persulfuric acid.

In oxidative esterifications of aldehyde **3** with *in situ*-generated persulfuric acid (see Scheme 3B in the manuscript), small amounts of acetal **7a** were detected as the only side product. In order to assign the HPLC signal of **7a**, a reference sample was prepared according to the following procedure.

A solution containing aldehyde **3** (1 eq, 0.1.0 M) and HCl (2 eq; HCl was used as 6.0 M aq solution) in MeOH was pumped at a flow rate of 500 µL min^{-1} by using a Syrris® Asia syringe pump equipped with an injection valve and a sample loop (15 mL; 1/16" OD, 0.80 mm ID) with MeOH serving as carrier solvent. The stream was directed through a 15-mL reaction coil (1/16" OD, 0.80 mm ID; 30 min residence time) which was heated at 100 °C in an oil bath. The system was pressurized at 5 bar by using a Zaiput BPR. The reactor outlet was directed into a flask containing saturated aq. $NaHCO_3$ and a magnetic stirrer. The product stream was collected for 10 min after reaching steady state. Then, the resulting mixture was extracted with CH_2Cl_2, washed with brine and dried over $MgSO_4$. The obtained material was analyzed by reversed-phase analytical HPLC, and also by ^{1}H NMR. The experiment yielded a reference sample of side product **7a** in >70% purity without any further work-up or purification.

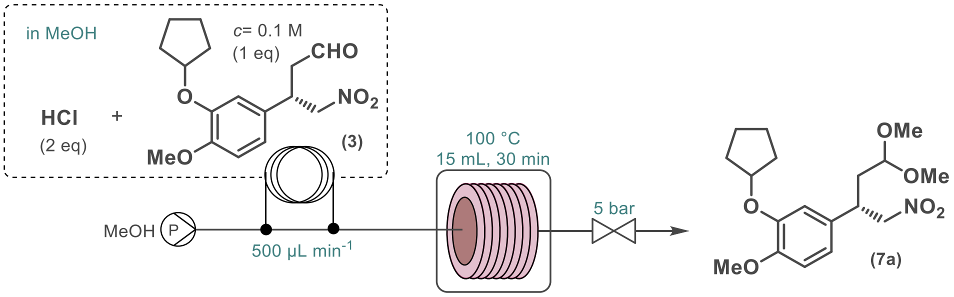

Scheme S9. Synthesis of side product **7a**.

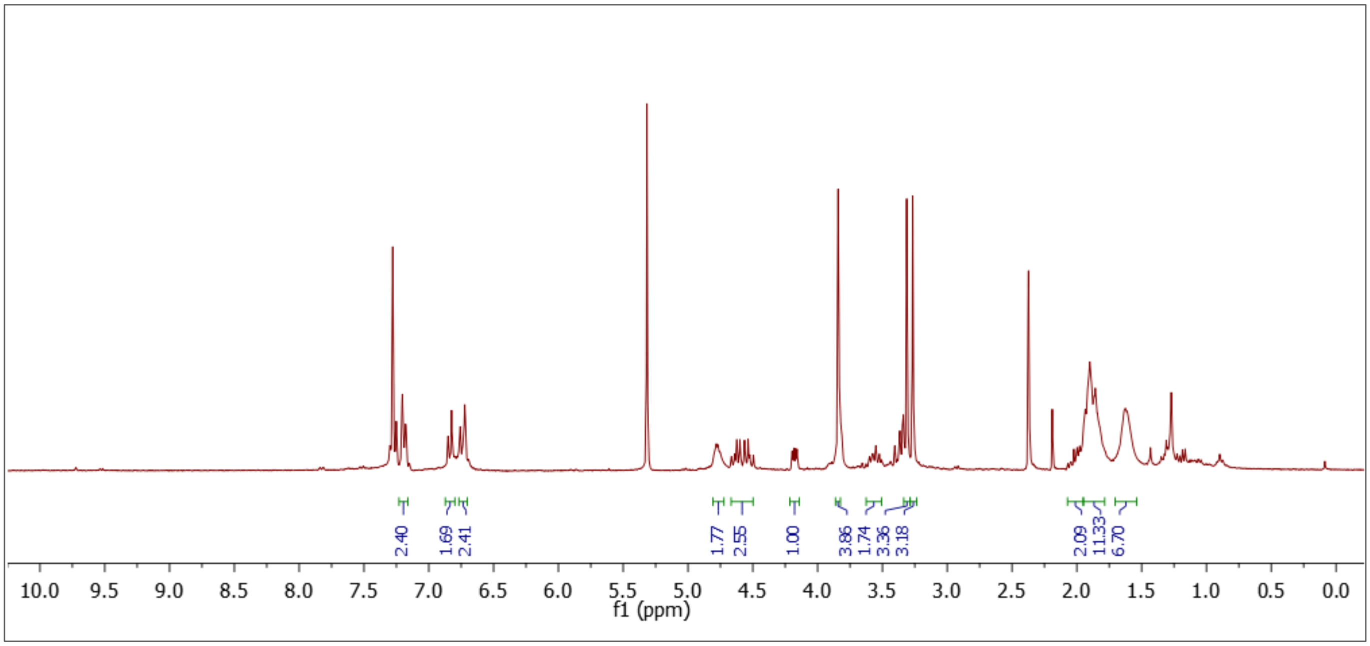

Figure S2. ^{1}H NMR spectrum (300 MHz, $CDCl_3$) of crude **7a**.

With the aim to facilitate telescoping of the first two reaction steps, a modified three-feed system was established and the persulfuric acid-mediated oxidative esterification of aldehyde **3** was performed according to the procedure described below.

Substrate solution containing aldehyde **3** (1.0 M) and a solution of H_2SO_4 (4.0 M; prepared from cc H_2SO_4) were pumped as separate streams (P1 and P2; both at 187 µL min^{-1}; corresponds to 1 eq of the substrate and 4 eq of the acid) by using Syrris® Asia syringe pumps equipped with two injection valves and two sample loops (SL1 and SL2: 8 mL; 1/16" OD, 0.80 mm ID, each). As a third stream, a solution of H_2O_2 in MeOH (2.0 M; prepared from 35 wt% aq. H_2O_2) was pumped by using a Syrris® Asia syringe pump (P3; at 375 µL min^{-1}; corresponds to 4 eq H_2O_2)

equipped with an injection valve and a sample loop (SL3: 15 mL; 1/16” OD, 0.80 mm ID). With MeOH serving as carrier solvent, the substrate and H_2SO_4 feeds were combined in a Y-mixer, then the resulting stream was mixed up with the H_2O_2 feed through a second Y-mixer. The combined liquid stream was next directed through a 15-mL reaction coil (1/16” OD, 0.80 mm ID; 20 min residence time) which was heated at 100 °C in an oil bath. The system was pressurized at 5 bar by using a Zaiput BPR. The reactor outlet was directed into a flask containing a stirred mixture of saturated aq. $NaHCO_3$ and some MnO_2 in order to quench any excess oxidant. The product stream was collected for 5 min after reaching steady state. Then, the resulting mixture was extracted with CH_2Cl_2, washed with brine and dried over $MgSO_4$. After evaporation, the obtained material was analyzed by reversed-phase analytical HPLC. Similarly to the reaction performed in the two-feed system under otherwise identical conditions (see Scheme 3B in the manuscript), quantitative conversion and 98% selectivity was obtained.

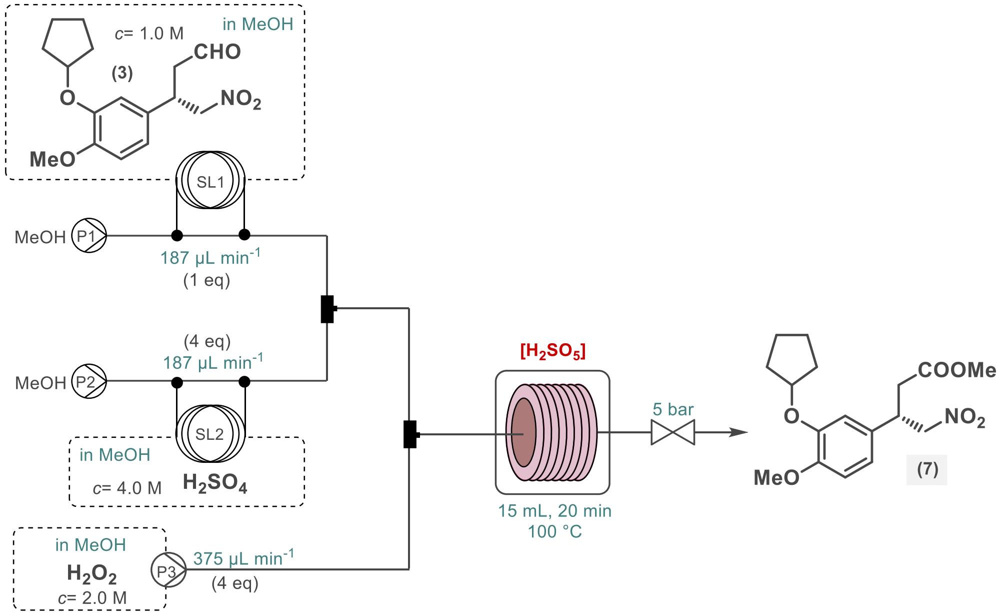

Scheme S10. Three-feed flow setup for the oxidative esterification of aldehyde **3** with *in situ*-generated persulfuric acid.

6. Experimental procedure for the telescoped flow synthesis of chiral ester 7

For the organocatalytic conjugate addition, 1.5 g of catalyst **2** was filled into an adjustable Omnifit® glass column (10 mm ID) which was heated by a Syrris® Asia column heater at 65 °C. A reaction mixture consisting of aldehyde **1** (1.0 M, 1 eq), CH_3NO_2 (5 eq) and AcOH (0.6 eq) in MeOH as solvent was introduced by using a Syrris® Asia syringe pump (P1) equipped with an injection valve and a sample loop (25 mL; 1/8” OD, 1.58 mm ID) with MeOH/EtOAc 1:1 serving as the carrier solvent. The flow rate was set to 100 µL min^{-1} which corresponded to 15 min residence time on the catalyst bed. The reaction mixture exiting the chiral catalyst column was directed through a 3-port valve, and a check valve was also installed to prevent any incidental backflow towards the catalyst column. Prior to the telescoped reaction, the catalyst was swollen by pumping EtOAc at 200 µL min^{-1} for 30 min, while the liquid stream exiting the column was directed to the waste through the three-port valve. 4.0 M H_2SO_4 solution (prepared from cc H_2SO_4) and 2.0 M H_2O_2 solution (prepared from 35 wt% aq. H_2O_2), both in MeOH as solvent, were introduced directly as separate feeds (P2 and P3) by using Syrris® Asia syringe pumps at flow rates of 100 µL min^{-1} and 200 µL min^{-1}, respectively, which corresponded to 4 eq of both reagents with respect to the aldehyde stream. The substrate stream exiting the catalyst column and the H_2SO_4 feed were combined in a Y-mixer, and the resulting stream was mixed up with the H_2O_2 feed through a second Y-mixer. The combined liquid stream was finally directed through an 8-mL reaction coil (1/16” OD, 0.80 mm ID; 20 min residence time) which was heated at 100 °C in an oil bath. The system was pressurized at 5 bar by using a Zaiput BPR after the heated coil and also by using an IDEX BPR at the switchable outlet after the catalyst column. The reactor outlet was directed into a flask containing a stirred mixture of saturated aq. $NaHCO_3$ and some MnO_2 in order to quench any excess oxidant.

In a typical experiment, the carrier solvent flow (P1) was started first, while the liquid stream exiting the column was directed to the waste through the 3-port valve. Once pressure and temperature were stabilized, the substrate stream was injected. 20 minutes after that, the H_2SO_4 and the H_2O_2 streams were also started. Before combining the two reactor segments, reference samples were taken from the conjugate addition stream for off-line GC-FID analysis. When the stream exiting the chiral catalyst column reached steady state (approx. 30 min after injecting the substrate solution), the flow direction was switched by the 3-port valve to initiate the esterification. The product stream exiting the heated reaction coil was collected continuously for 3 h after reaching steady state (30 min after switching the 3-port valve). To finish the experiment, the 3-port valve was switched back to waste direction, substrate injection was stopped, and H_2SO_4 and H_2O_2 streams were replaced with MeOH as washing solvent. The catalyst bed was washed carefully with EtOAc/AcOH 9:1 (0.5 mL min^{-1}, 30 min) then with EtOAc (0.2 mL min^{-1}, 45 min).

The resulting material was extracted with CH_2Cl_2, washed with brine and dried over $MgSO_4$. After evaporation, the obtained material was analyzed by reversed-phase analytical HPLC, and also by NMR. The experiment yielded 5.23 g (86%) of chiral ester **7** in a sufficiently pure form without any further work-up or chromatographic purification. The process ensured a productivity of 1.74 g h^{-1}. The ee of the material was 94%.

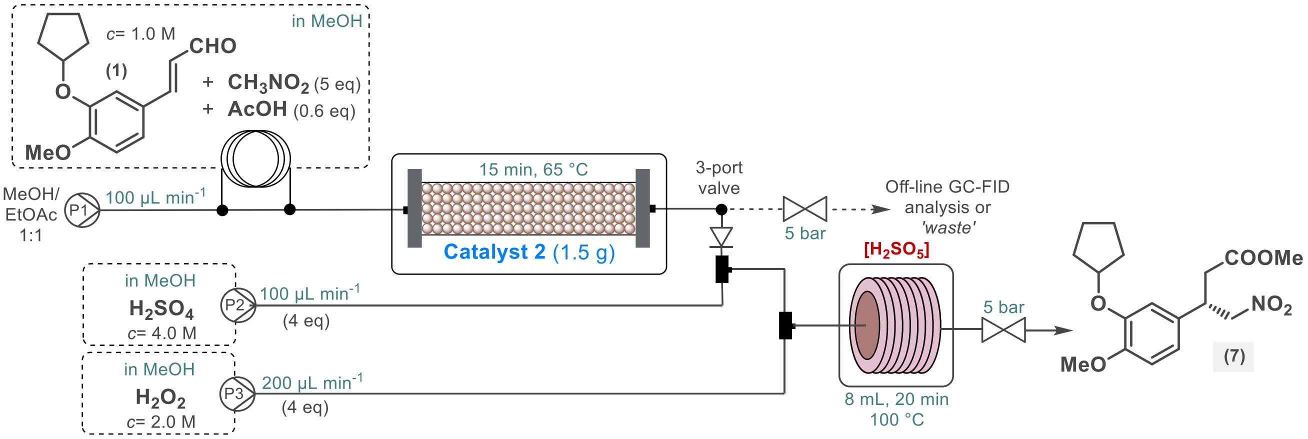

Scheme S11. Telescoped organocatalytic conjugate addition–oxidative esterification sequence for the synthesis of nitroester **7**.

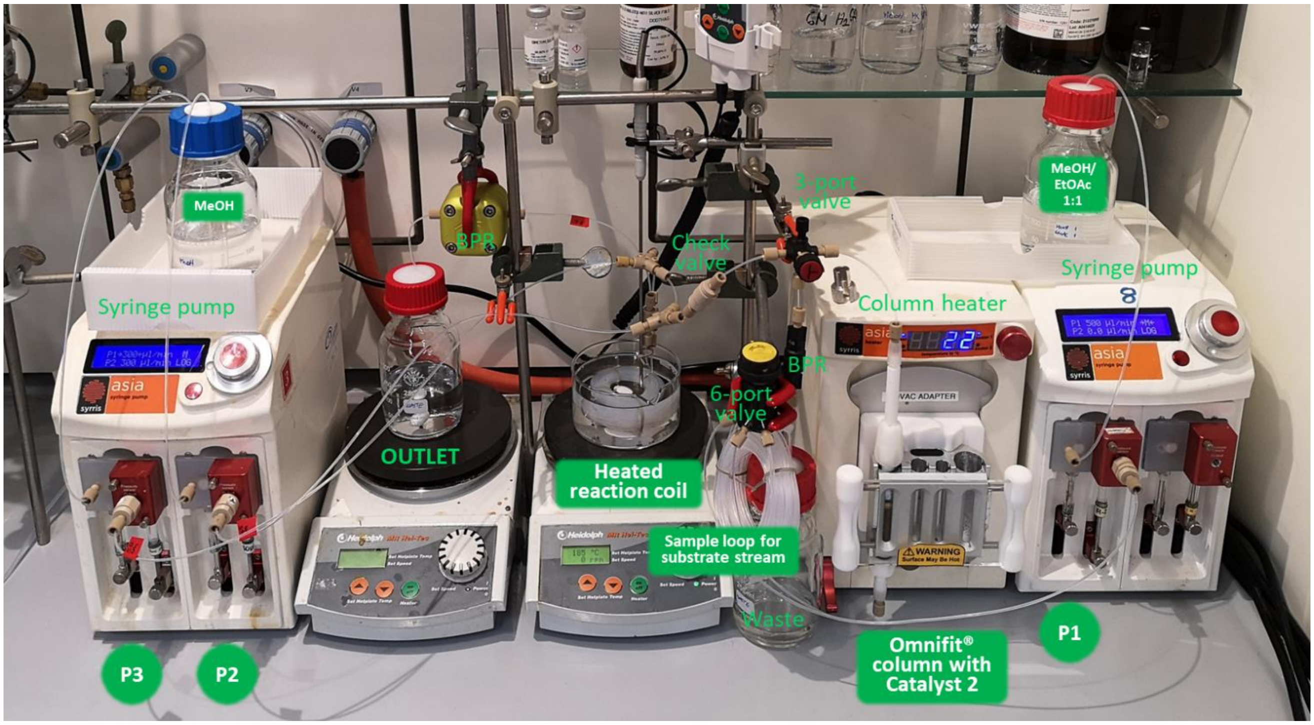

Figure S3. A photograph of the telescoped flow system.

7. Experimental procedure for the nitro reduction/lactamization

Substrate solution containing ester **7** (1 eq, 0.4 M) together with DIEA or TEA (3, 4, 4.5 or 6 eq) in dry CH_2Cl_2, CH_3CN or CH_3CN/CH_2Cl_2 7:1 as solvent and a solution of $HSiCl_3$ (1.2 or 1.6 M) in dry CH_2Cl_2 or CH_3CN were pumped as separate streams (P1 and P2; both at 50 or 100 µL min^{-1}) by using Syrris® Asia syringe pumps equipped with two injection valves and two sample loops (SL1 and SL2: 3 mL; 1/16” OD, 0.80 mm ID, each). With dry CH_2Cl_2 or CH_3CN serving as carrier solvent, the feeds were combined in a Y-mixer. The resulting stream was directed through a 1.0-mL reaction coil (1/16” OD, 0.80 mm ID; 5 or 10 min residence time) at room temperature. The system was pressurized at 5 bar by using a Zaiput BPR. In order to quench the reaction, the reactor outlet was directed into a flask containing 2.0 M aq NaOH solution and a magnetic stirrer. The product stream was collected for 20 min after reaching steady state. Then, the resulting mixture was extracted with EtOAc, washed with brine and dried over Na_2SO_4. After evaporation, the obtained material was analyzed by reversed-phase analytical HPLC, and also by ^{1}H NMR. See Scheme 5 in the manuscript for the corresponding reaction data.

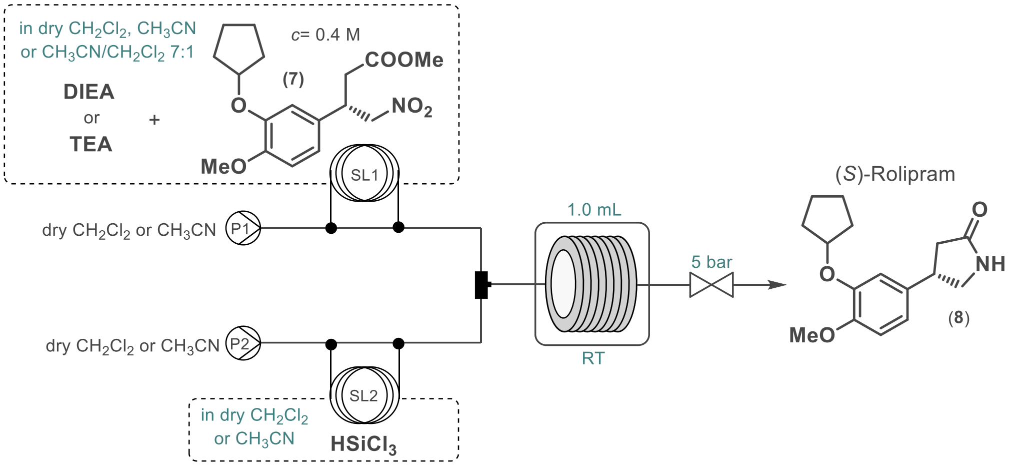

Scheme S12. Continuous flow setup for nitro reduction/lactamization of **7** in the presence of $HSiCl_3$.

For the long-run (see Scheme 5 in the manuscript), a similar reaction setup was used. Substrate solution containing ester **7** (1 eq, 0.4 M) together with DIEA (4 eq) in dry CH_3CN/CH_2Cl_2 7:1 as solvent and a solution of $HSiCl_3$ (1.6 M) in dry CH_3CN were introduced as separate streams (P1 and P2) using two sample loops (SL1 and SL2: 14 mL; 1/16” OD, 0.80 mm ID, each). CH_3CN served as carrier solvent. Both flow rates were set to 50 µL min^{-1}, which corresponded 10 min residence time (1.0-mL reaction coil, room temperature). The reactor outlet was directed into a flask containing 2.0 M aq NaOH solution and a magnetic stirrer. The product stream was collected for 4 h after reaching steady state. Then, the resulting mixture was extracted with EtOAc, washed with brine and dried over Na_2SO_4. After evaporation, the obtained material was analyzed by reversed-phase analytical HPLC, and also by ^{1}H NMR. Finally, after column chromatographic purification (a mixture of EtOAc/40-60 petroleum ether was used as eluent), 1.10 g (83%) of (*S*)-rolipram (**8**) was isolated. The ee of the material was 94%.

8. Preparation of racemic reference samples

A racemic reference sample of γ-nitroaldehyde **3** was prepared by using an 1:1 mixture of (*R*)- and (S)-α,α-diphenyl-2-pyrrolidinemethanol trimethylsilyl ether as organocatalyst according to the following procedure. A mixture containing α,β-unsaturated aldehyde **1** (9 mmol), nitromethane (27 mmol), AcOH (2.7 mmol) and 10 mol% of the racemic catalyst was stirred for 24 h at RT in MeOH (20 mL) as solvent. After then, the mixture was filtrated through a pad of celite, washed with CH_2Cl_2 and evaporated. The crude product was purified by means of column chromatography using a mixture of ethyl acetate/40-60 petroleum ether as eluent. Racemic samples of ester **7** and rolipram (**8**) were prepared from (*rac*)-**3** by oxidative esterification followed by nitro reduction/lactamization according to the general continuous flow procedures detailed in sections 5.2 and 7.

9. Characterization data

(*E*)-3-(3-(Cyclopentyloxy)-4-methoxyphenyl)acrylaldehyde (**1**):

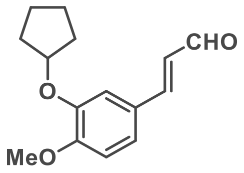

Following the procedure described in section 3, in a typical synthesis batch 7.75 g (31.46 mmol, 91%) of the title compound was obtained as a beige solid (mp: 78–80 °C).

NMR data of the compound matches the reported literature.[S11]

(*S*)-3-(3-(cyclopentyloxy)-4-methoxyphenyl)-4-nitrobutanal (**3**):

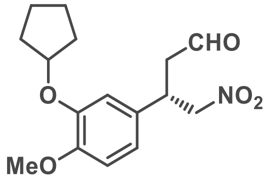

Following the general continuous flow procedure described in section 4, 11.48 g (37.35 mmol, 92%) of the title compound was obtained as yellowish oil.

NMR data of the compound matches the reported literature.[S15]

HRMS (ESI) calculated for $C_{16}H_{21}NO_5$: 307.1420, found: 307.1424.

A small sample of the product was derivatized into the corresponding methyl ester according to a literature procedure,[S11] and *ee* was determined by HPLC using a Chiralpak® IB column (hexane–*i*PrOH 94/6, 0.8 mL min^{-1}, 210 nm, 25 °C): t_{minor}= 16.00 min, t_{major}= 17.55 min, *ee*= 94%.

Methyl 3-phenylpropanoate (**4**):

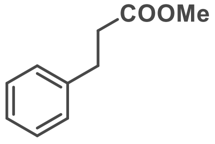

Following the general continuous flow procedure described in section 5.2, 93.4 mg (0.569 mmol, 91%) of the title compound was obtained as a colorless liquid.

NMR data of the compound matches the reported literature.[S16]

Methyl (*S*)-3-(3-(cyclopentyloxy)-4-methoxyphenyl)-4-nitrobutanoate (**7**):

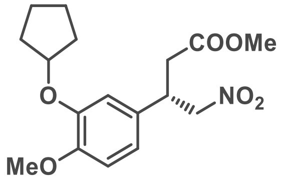

Following the general continuous flow procedure described in section 6, 5.23 g (15.50 mmol, 86%) of the title compound was obtained as a white solid (mp: 103–105 °C).

NMR data of the compound matches the reported literature.[S11]

HRMS (ESI) calculated for $C_{17}H_{23}NO_6$: 337.1525, found: 337.1514.

The *ee* was determined by HPLC using a Chiralpak® IB column (hexane–*i*PrOH 94/6, 0.8 mL min^{-1}, 210 nm, 25 °C): t_{minor}= 15.98 min, t_{major}= 17.50 min, *ee*= 94%. $[\alpha]_D^{25}$= –11.8 (c= 1.00, $CHCl_3$) {lit.[S11] $[\alpha]_D^{25}$= –18.3 (c= 1.00, $CHCl_3$)}.

(*S*)-Rolipram (**8**):

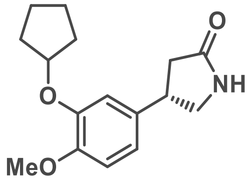

Following the general continuous flow procedure described in section 7, 1.10 g (4.00 mmol, 83%) of the title compound was obtained as a white solid (mp: 133–136 °C; lit.[S17] 129–132 °C).

NMR data of the compound matches the reported literature.[S11]

HRMS (ESI) calculated for $C_{16}H_{21}NO_3$: 275.1521, found: 275.1513.

The *ee* was determined by HPLC using a Chiralpak® IA column (hexane–*i*PrOH 95/5, 1 mL min^{-1}, 210 nm, 25 °C): t_{minor}= 22.88 min, t_{major}= 23.86 min, *ee*= 94%. $[\alpha]_D^{25}$= +27.4 (c= 0.135, $CHCl_3$) {lit.[S18] $[\alpha]_D^{25}$= +28.3 (c= 0.135, $CHCl_3$)}.

10. Collection of NMR spectra and HPLC chromatograms

^{1}H-NMR
(300 MHz, $CDCl_3$)

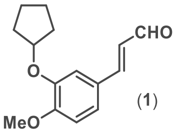

(1)

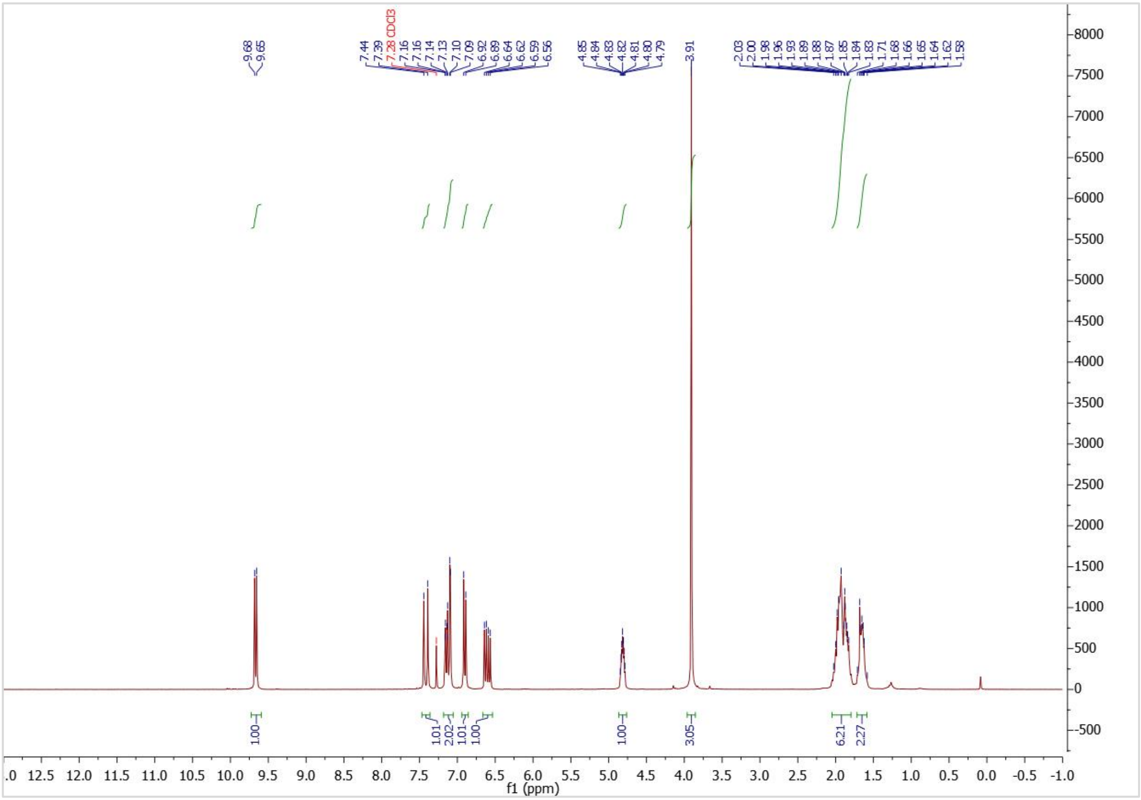

^{13}C-NMR
(75 MHz, $CDCl_3$)

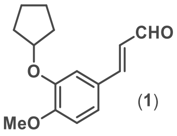

(1)

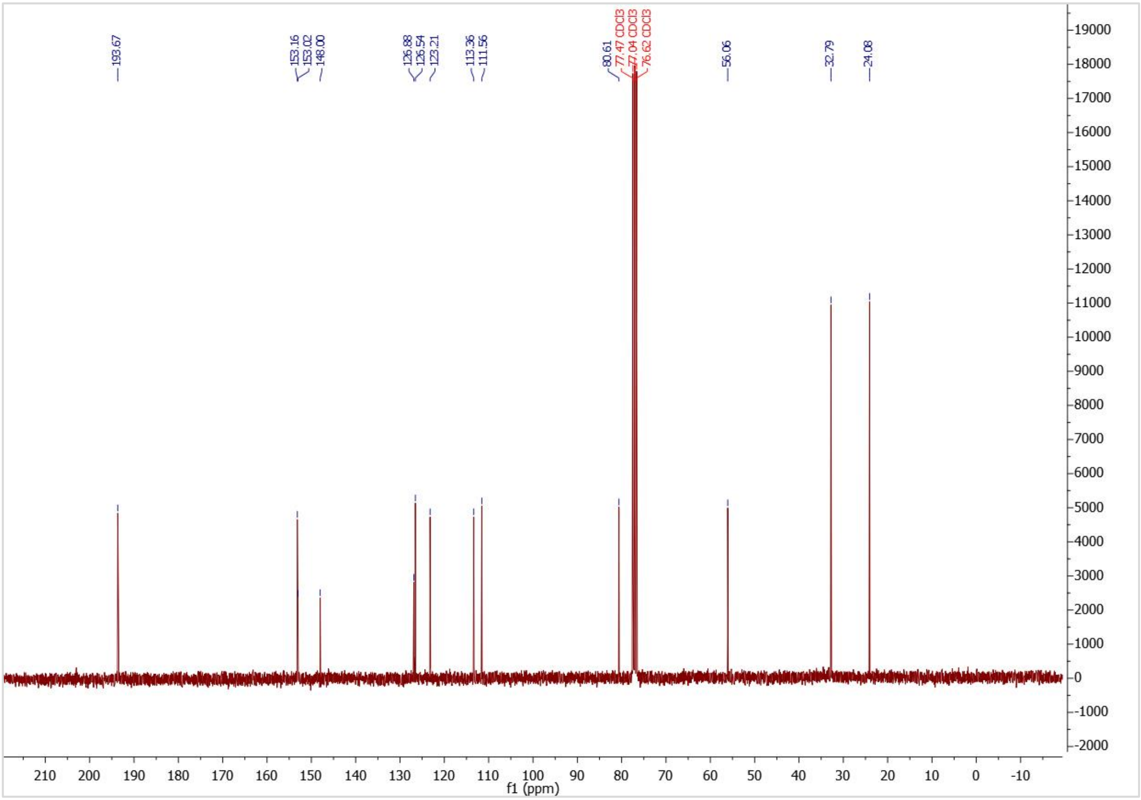

^{1}H-NMR
(300 MHz, $CDCl_3$)

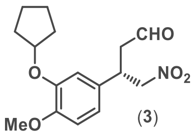

(3)

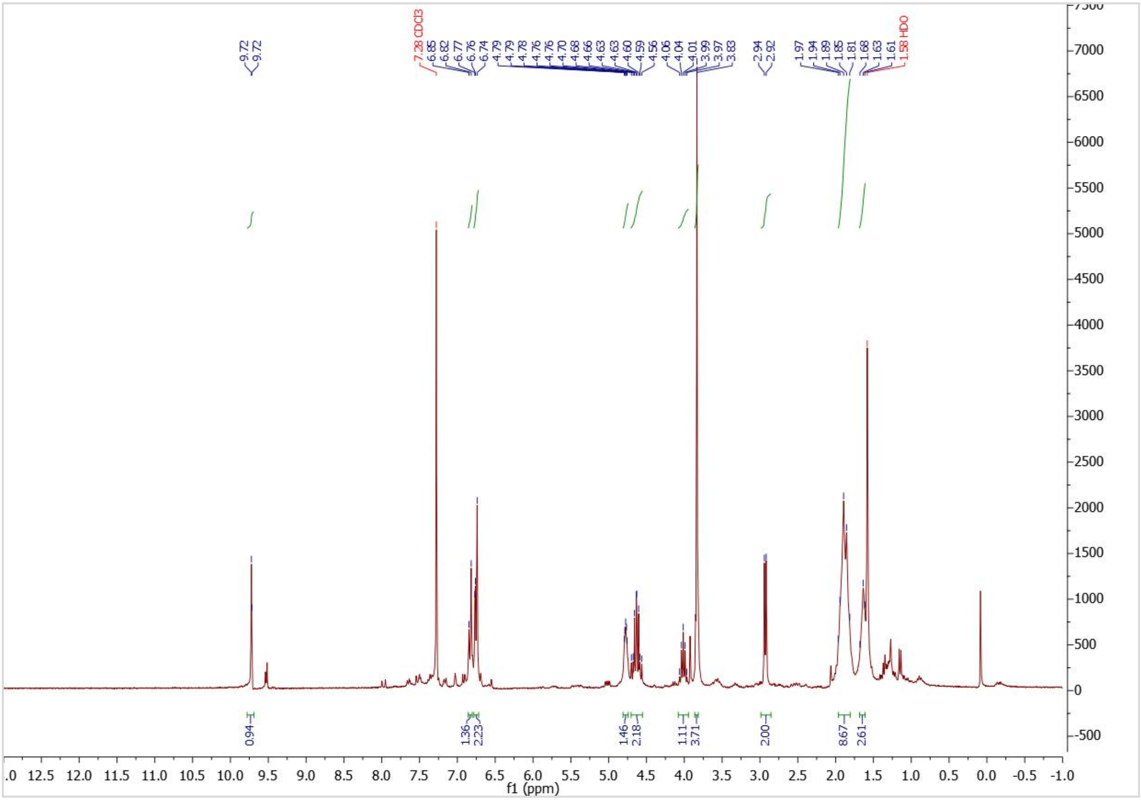

^{13}C-NMR
(75 MHz, $CDCl_3$)

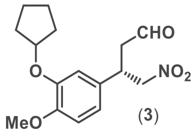

(3)

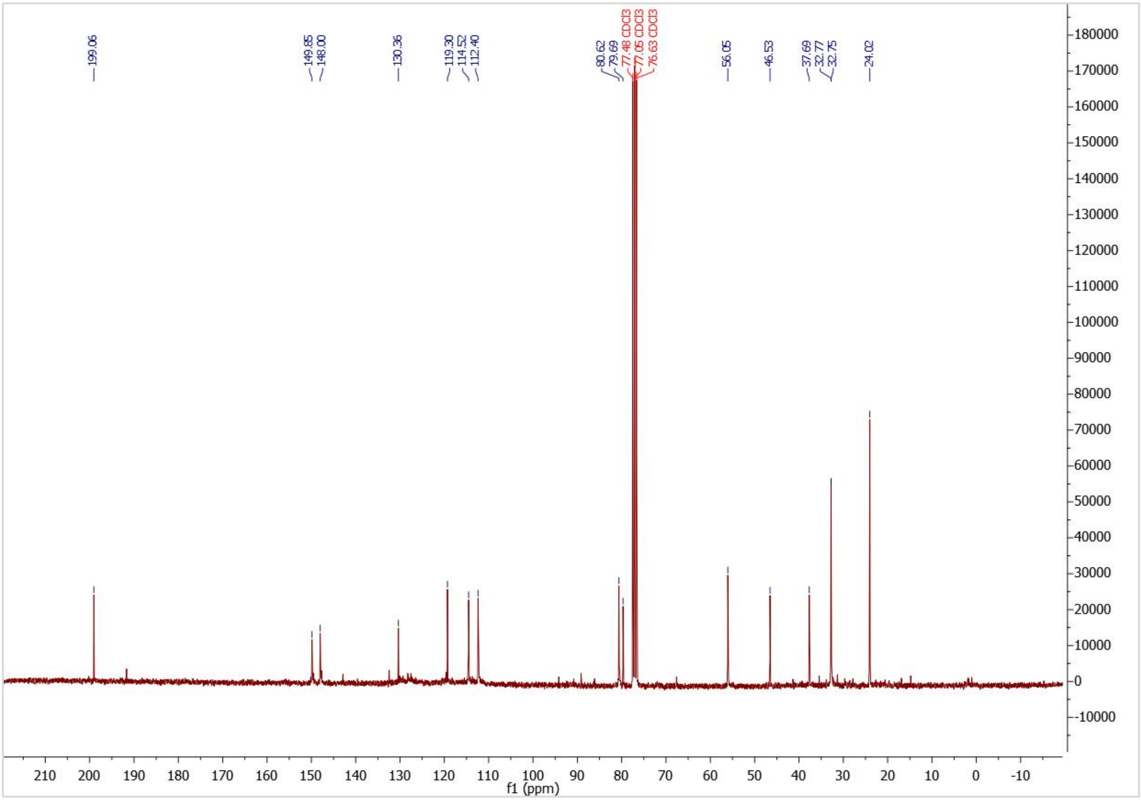

HPLC chromatograms

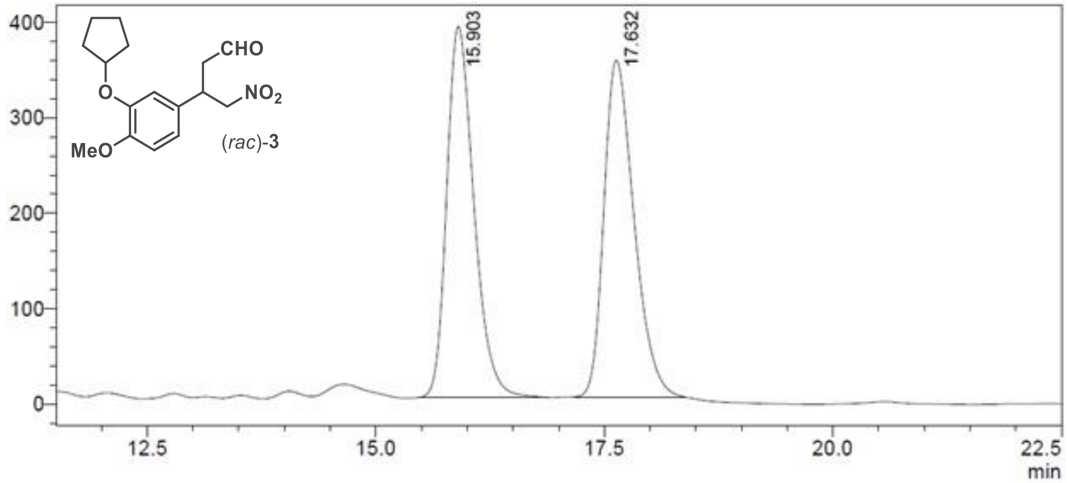

Peak#	Ret. Time	Area	Height	Area%
1	15.903	8059558	389410	49.870
2	17.632	8101615	353413	50.130
Total		16161173	742823	100.000

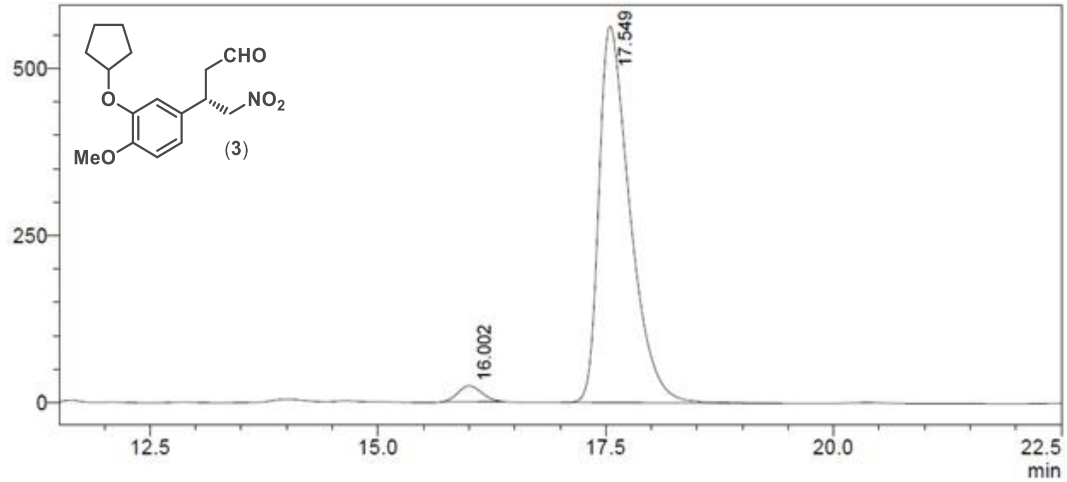

Peak#	Ret. Time	Area	Height	Area%
1	16.002	438729	24022	3.186
2	17.549	13332270	562898	96.814
Total		13771000	586921	100.000

^{1}H-NMR
(300 MHz, $CDCl_3$)

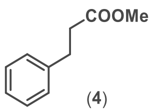

(**4**)

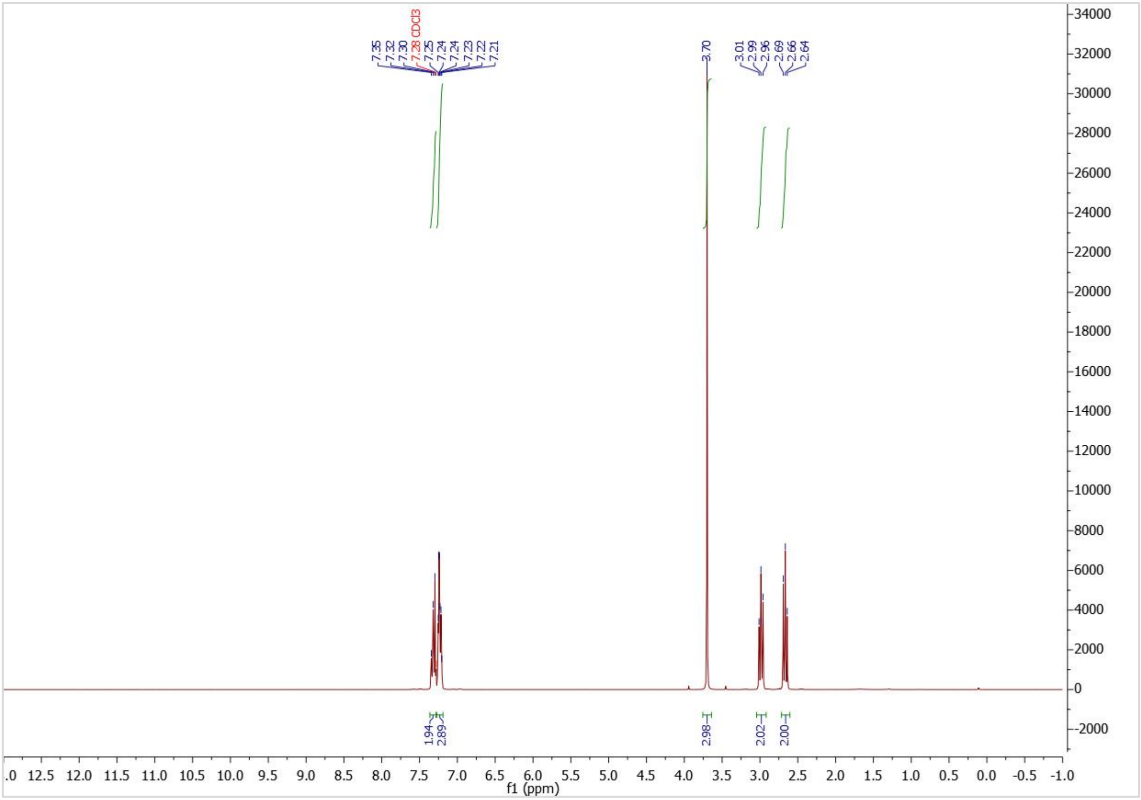

^{13}C-NMR
(75 MHz, $CDCl_3$)

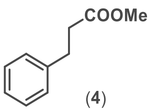

(**4**)

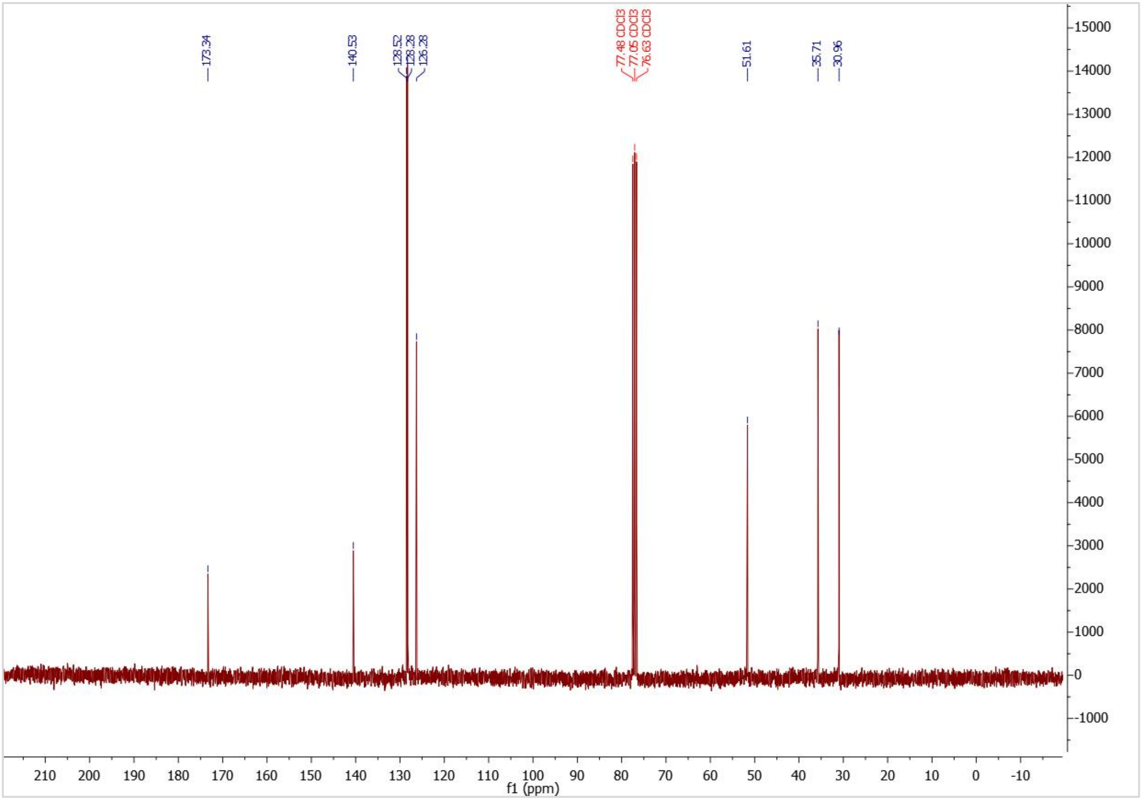

^{1}H-NMR
(300 MHz, $CDCl_3$)

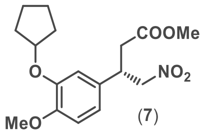

(7)

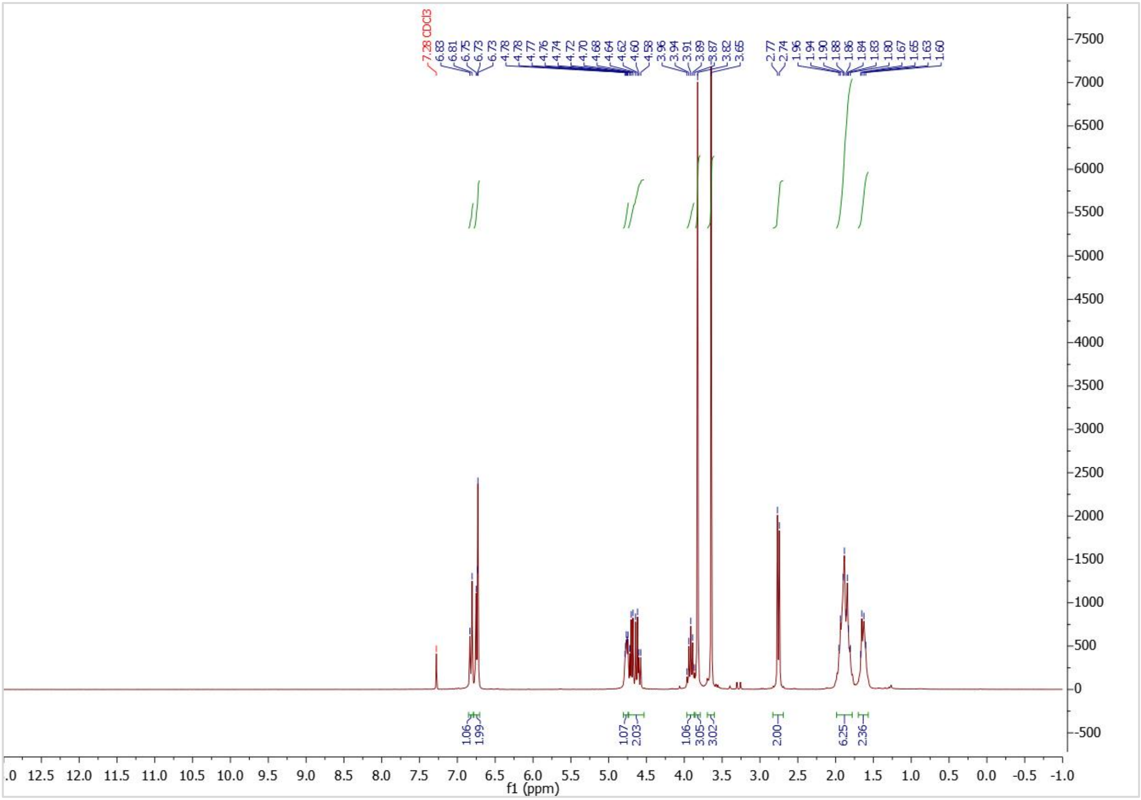

^{13}C-NMR
(75 MHz, $CDCl_3$)

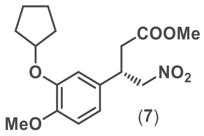

(7)

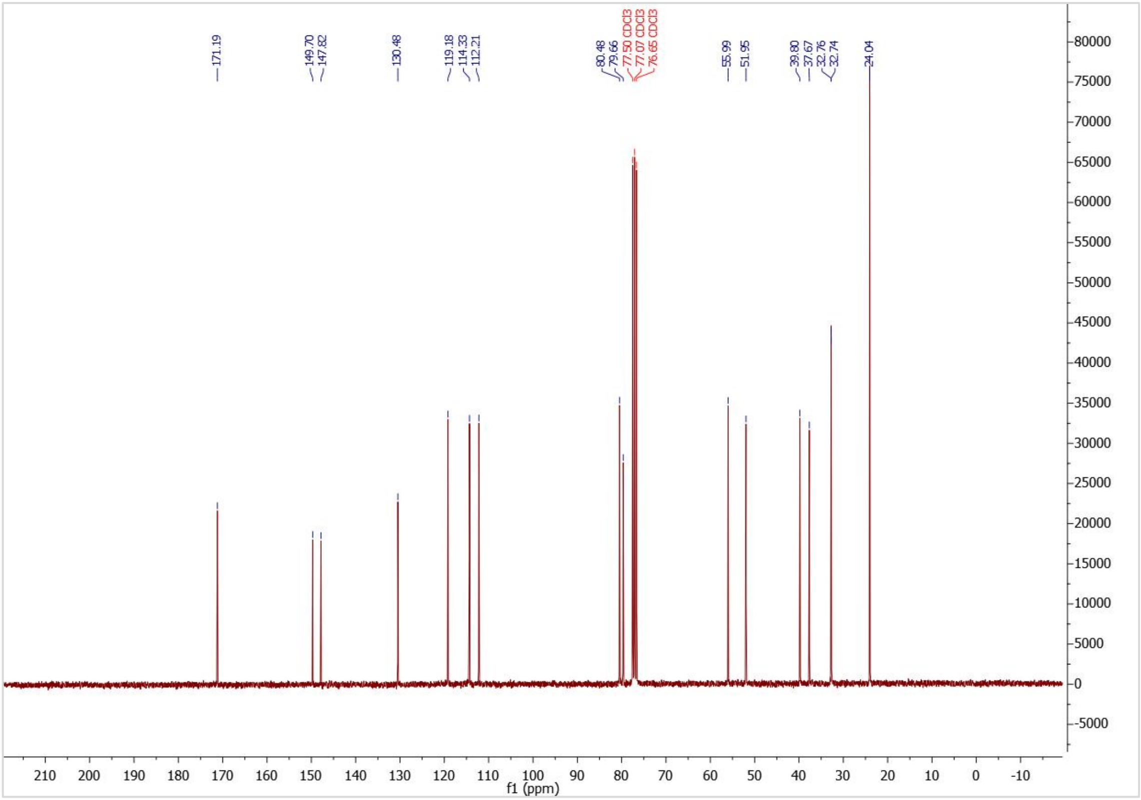

HPLC chromatograms

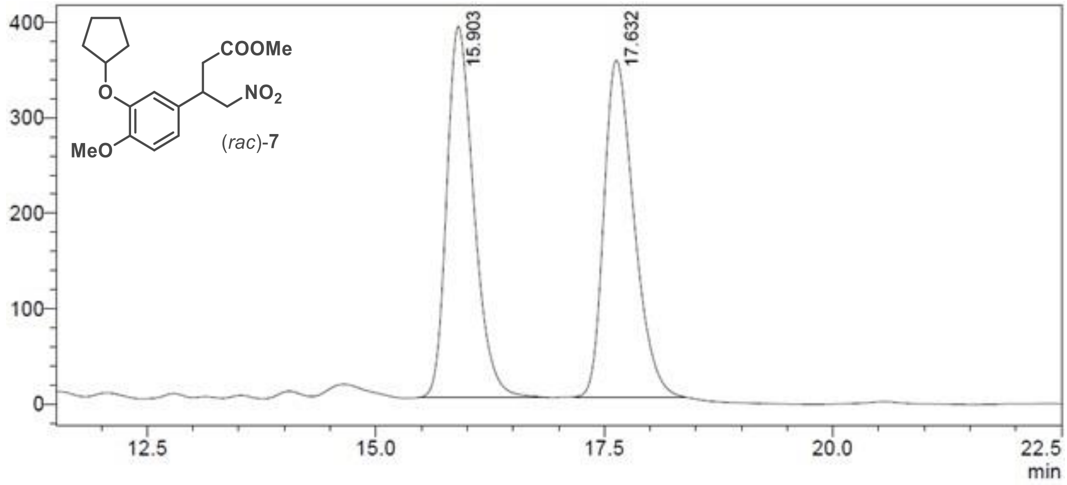

Peak#	Ret. Time	Area	Height	Area%
1	15.903	8059558	389410	49.870
2	17.632	8101615	353413	50.130
Total		16161173	742823	100.000

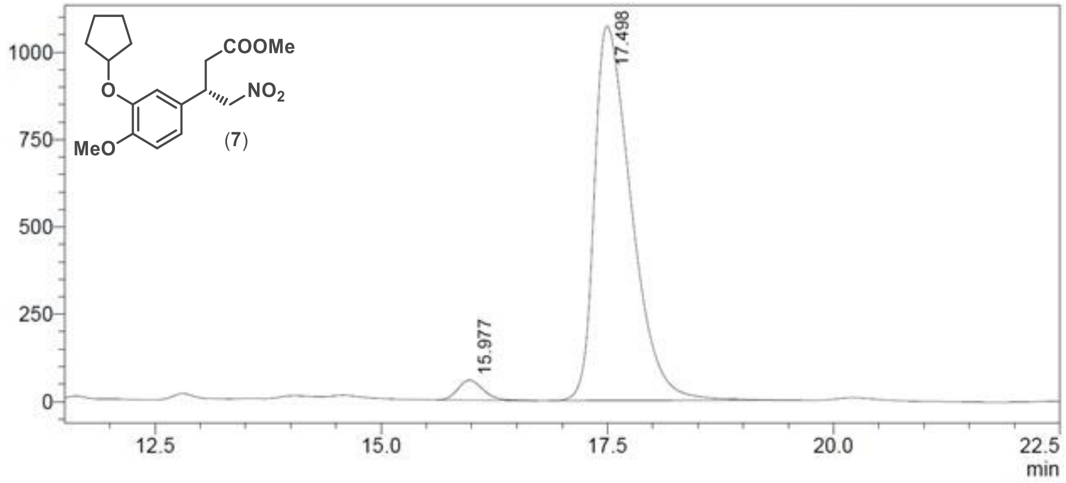

Peak#	Ret. Time	Area	Height	Area%
1	15.977	935201	53375	2.973
2	17.498	30518766	1072070	97.027
Total		31453967	1125444	100.000

^{1}H-NMR
(300 MHz, $CDCl_3$)

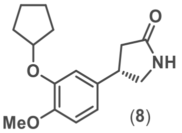

(**8**)

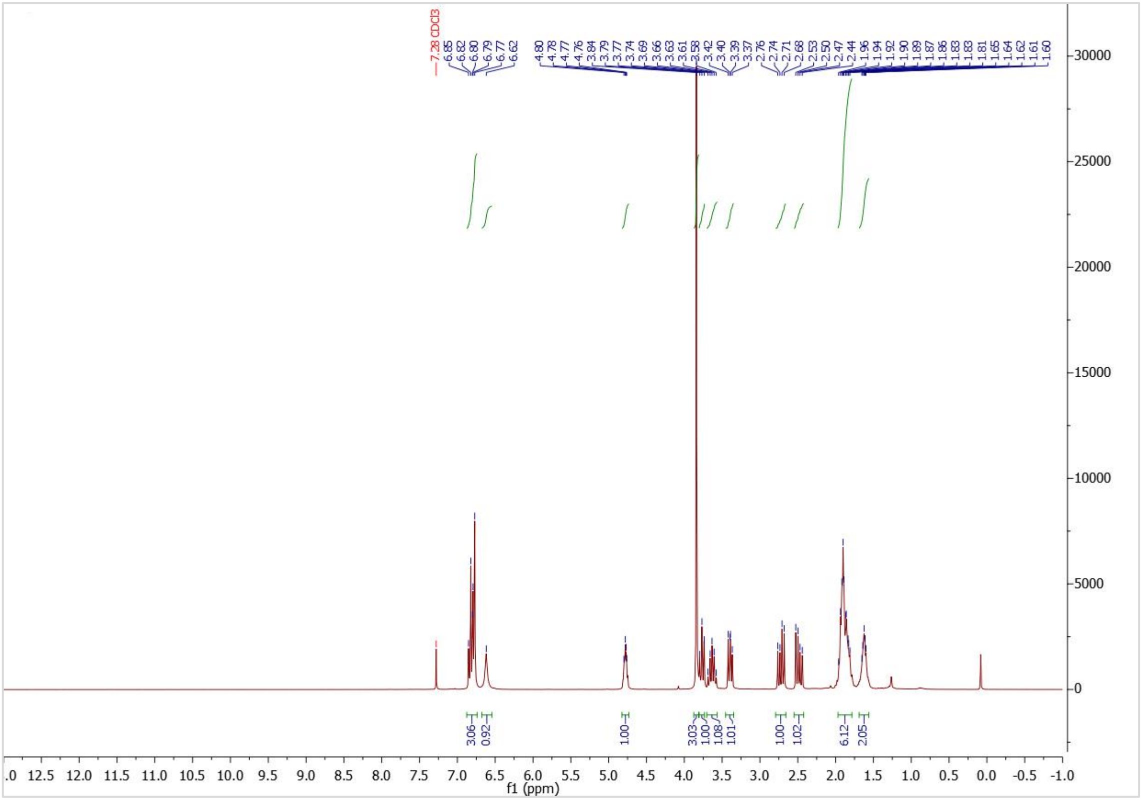

^{13}C-NMR
(75 MHz, $CDCl_3$)

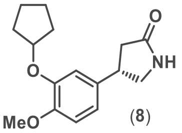

(**8**)

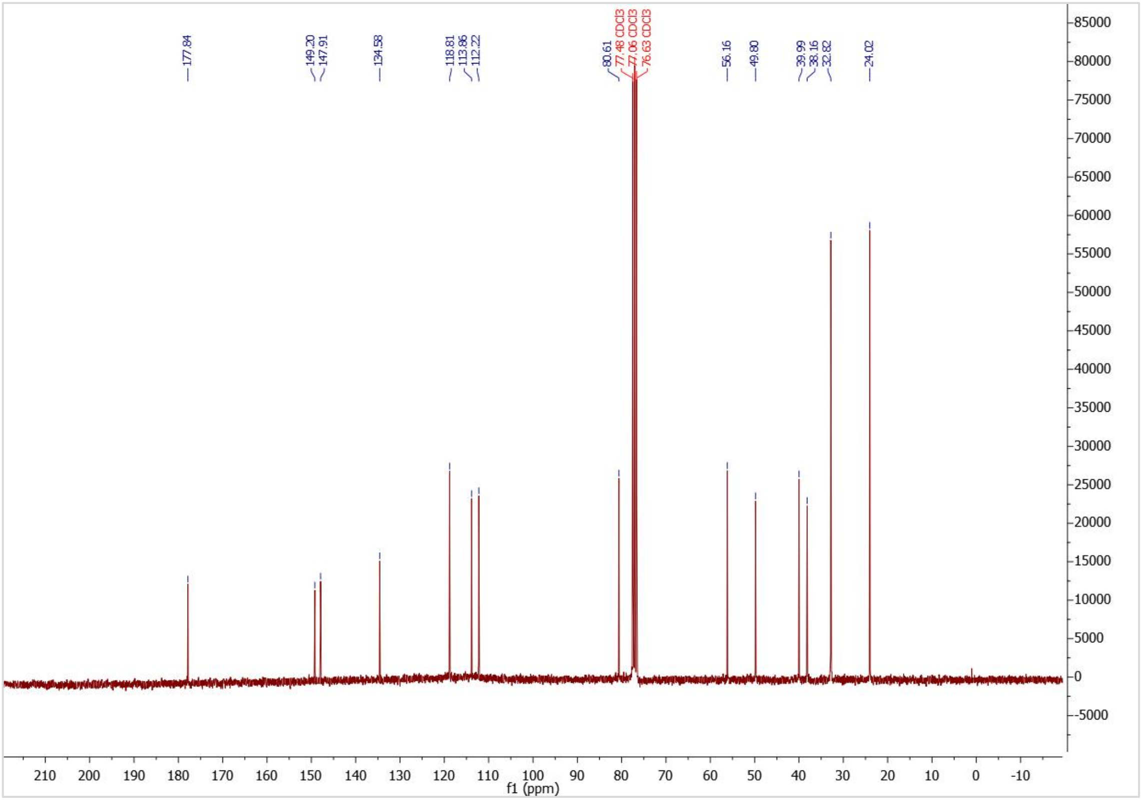

HPLC chromatograms

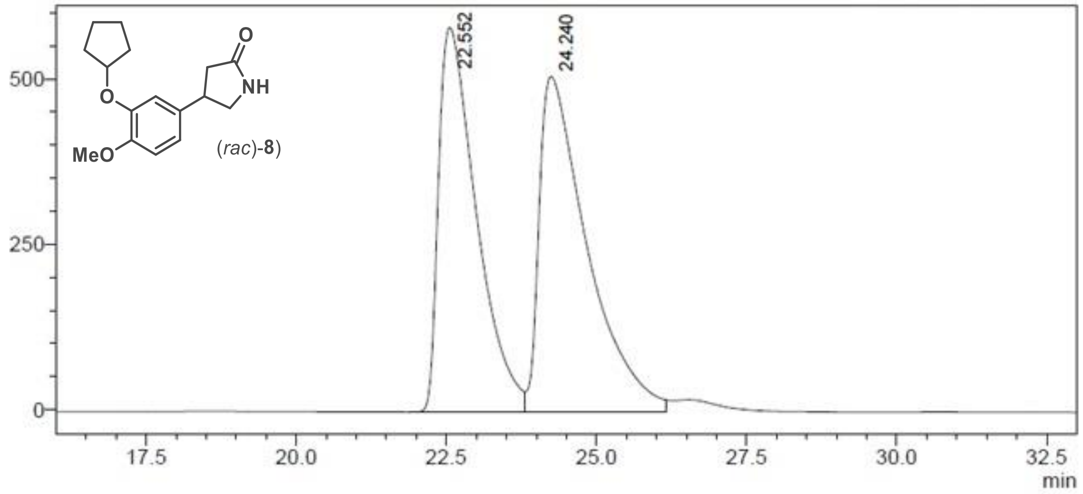

Peak#	Ret. Time	Area	Height	Area%
1	22.552	25490455	581603	47.439
2	24.240	28242806	507476	52.561
Total		53733261	1089080	100.000

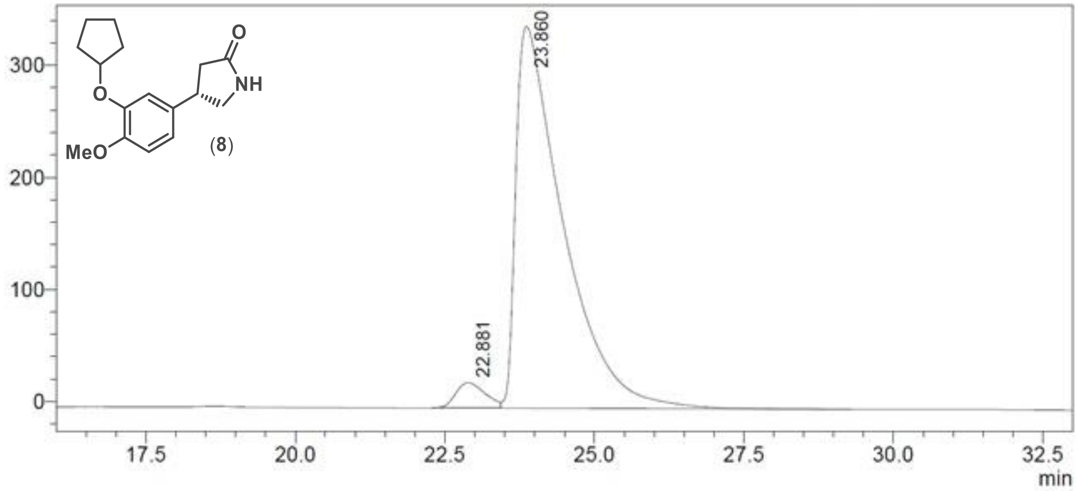

Peak#	Ret. Time	Area	Height	Area%
1	22.881	552446	19174	3.208
2	23.860	16666676	328462	96.792
Total		17219122	347636	100.000

11. References

(S1) I. Arenas, A. Ferrali, C. Rodriguez-Escrich, F. Bravo, M. A. Pericas, *Adv. Synth. Catal.* **2017**, *359*, 2414-2424.

(S2) J. Lai, S. Sayalero, A. Ferrali, L. Osorio-Planes, F. Bravo, C. Rodriguez-Escrich, M. A. Pericas, *Adv. Synth. Catal.* **2018**, 360, 2914-2924.

(S3) J. Izquierdo, C. Ayats, A. H. Henseler, M. A. Pericas, *Org. Biomol. Chem.* **2015**, *13*, 4204-4209.

(S4) S. B. Ötvös, M. A. Pericàs, C. O. Kappe, *Chem. Sci.* **2019**, *10*, 11141-11146.

(S5) S. B. Ötvös, P. Llanes, M. A. Pericàs, C. O. Kappe, *Org. Lett.* **2020**, *22*, 8122-8126.

(S6) J. J. Conde: *Process and Intermediates for Making 4-Cyanosubstituted Cyclohexanoic Acids*, WO 01/19785.

(S7) B. K. Wilk, N. Mwisiya, J. L. Helom, *Org. Process Res. Dev.* **2008**, *12*, 785-786.

(S8) G. Battistuzzi, S. Cacchi, G. Fabrizi, *Org. Lett.* **2003**, *5*, 777-780.

(S9) J. J. Molloy, J. B. Metternich, C. G. Daniliuc, A. J. B. Watson, R. Gilmour, *Angew.Chem. Int. Ed.* **2018**, *57*,3168-3172.

(S10) Y Takashima, Y. Isogawa, A. Tsuboi, N. Ogawa, Y. Kobayashi, *Org. Biomol. Chem.*, **2021**,*19*, 9906-9909.

(S11) C. Palomo, A. Landa, A. Mielgo, M. Oiarbide, Ángel Puente, S. Vera, *Angew. Chem. Int. Ed.* **2007**, *46*, 8431-8435.

(S12) M. Prieschl, S. B. Ötvös, C. O. Kappe, *ACS Sustainable Chem. Eng.* **2021**, *9*, 5519-5525.

(S13) N. Spiliopoulou, N. F. Nikitas, C. G. Kokotos, *Green Chem.* **2020**, *22*, 3539-3545.

(S14) K. Žmitek, M. Zupan, S. Stavber, J. Iskra, *J. Org. Chem.* **2007**, *72*, 17, 6534-6540.

(S15) P. García-García, A. Ladépêche, R. Halder, B. List, *Angew. Chem. Int. Ed.* **2008**, *47*, 4719-4721.

(S16) T. Ikeda, Z. Zhang, Y. Motoyama, *Adv. Synth. Catal.* **2019**, *361*, 673-677.

(S17) P. S. Hynes, P. A. Stupple, D. J. Dixon, Org. Lett. **2008**, *10*, 1389-1391.

(S18) J. Ma, Q. Zhou, G. Song, Y. Song, G. Zhao, K. Ding, B. Zhao, *Angew. Chem. Int. Ed.* **2021**, *60*, 10588-10592.
